# Structure and Absolute Configuration of Phenanthro-perylene Quinone Pigments from the Deep-Sea Crinoid *Hypalocrinus naresianus*

**DOI:** 10.3390/md19080445

**Published:** 2021-08-03

**Authors:** Sahithya Phani Babu Vemulapalli, Juan Carlos Fuentes-Monteverde, Niels Karschin, Tatsuo Oji, Christian Griesinger, Klaus Wolkenstein

**Affiliations:** 1 Department of NMR-Based Structural Biology, Max Planck Institute for Biophysical Chemistry, Am Fassberg 11, 37077 Göttingen, Germany; save@nmr.mpibpc.mpg.de (S.P.B.V.); jufu@nmr.mpibpc.mpg.de (J.C.F.-M.); nkar@nmr.mpibpc.mpg.de (N.K.); 2 Institute for Chemistry and Biology of the Marine Environment, University of Oldenburg, Carl-von-Ossietzky-Str. 9-11, 26129 Oldenburg, Germany; 3 Nagoya University Museum, Nagoya University, Nagoya 464-8601, Japan; oji@num.nagoya-u.ac.jp; 4 Department of Geobiology, Geoscience Centre, University of Göttingen, Goldschmidtstraße 3, 37077 Göttingen, Germany

**Keywords:** crinoid, *Hypalocrinus naresianus*, phenanthroperylene quinones, gymnochromes, configuration determination

## Abstract

Two new water-soluble phenanthroperylene quinones, gymnochrome H (**2**) and monosulfated gymnochrome A (**3**), as well as the known compounds gymnochrome A (**4**) and monosulfated gymnochrome D (**5**) were isolated from the deep-sea crinoid *Hypalocrinus naresianus*, which had been collected in the deep sea of Japan. The structures of the compounds were elucidated by spectroscopic analysis including HRMS, 1D ^1^H and ^13^C NMR, and 2D NMR. The absolute configuration was determined by ECD spectroscopy, analysis of *J*-couplings and ROE contacts, and DFT calculations. The configuration of the axial chirality of all isolated phenanthroperylene quinones (**2**–**5**) was determined to be (*P*). For gymnochrome H (**2**) and monosulfated gymnochrome A (**3**), a (2′*S*,2″*R*) configuration was determined, whereas for monosulfated gymnochrome D (**5**) a (2′*R*,2″*R*), configuration was determined. Acetylated quinones are unusual among natural products from an echinoderm and gymnochrome H (**2**) together with the recently reported gymnochrome G (**1**) represent the first isolated acetylated phenanthroperylene quinones.

## 1. Introduction

Recently, a series of amido- and aminoanthraquinones, amidoanthraquinone biaryls, as well as non-nitrogen-containing anthraquinones have been isolated from the deep-sea crinoid *Hypalocrinus naresianus* collected off Japan in a water depth of 763 to 852 m [1]. Preliminary analysis of an ethanolic extract of one specimen also indicated the presence of a series of brominated phenanthroperylene quinones [2]. The structure of one of these phenanthroperylene quinones, gymnochrome G (**1**), has been described elsewhere [3].

While the determination of the absolute configuration of the phenanthroperylene quinone moiety is straightforward from ECD [4] due to the inherent axial chirality of sterically hindered phenanthroperylene quinone chromophores, the determination of configuration of chiral side chains at the chromophore needs other methods than ECD. As the chromophore can be used as chiral reference, these other methods need to be only diastereodiscriminating, such as NMR. In their impressive work, De Riccardis et al. [5] have determined the configuration of phenanthroperylene quinones from the stalked crinoid *Neogymnocrinus richeri*, named gymnochromes, by applying the empirical approach of Horeau [6] and using proton shielding effects established on the related cercosporin family [7]. The configuration of chiral centers of side chains of further gymnochromes from the stalked deep-water crinoid *Holopus rangii* have been tentatively assigned [8] using arguments similar to those of Nasini et al. [7] and De Riccardis et al. [5] based on the observation of shielding (or the lack of shielding) for the side chain protons.

In this study, we report the isolation, structure, and absolute configuration of those phenanthroperylene quinone pigments from *H*. *naresianus* that have been isolated in addition to gymnochrome G (**1**). We determined the configurations from *J*-couplings and ROE contacts and found that the empirical rules applied to the previously studied gymnochromes apply also to the gymnochromes described in this work.

## 2. Results and Discussion

**Figure 1:**
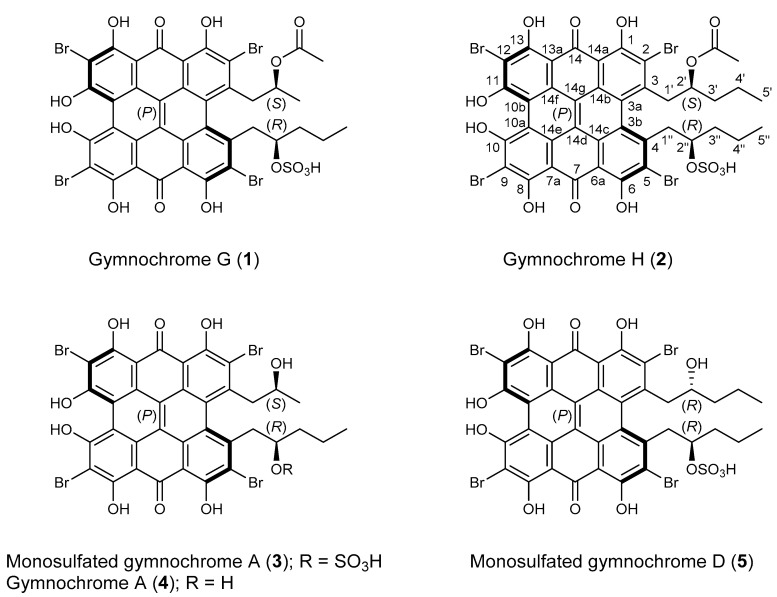
Chemical structures of gymnochromes isolated from *Hypalocrinus naresianus*.

The methanol-soluble pigments of the MeOH/CH_2_Cl_2_ extract of *H. naresianus* were combined with the MeOH/H_2_O (9:1), MeOH/H_2_O (1:1), and H_2_O extracts of the crinoid and subjected to semipreparative HPLC [1]. Fractions were then desalted by solid phase extraction to give the previously reported gymnochrome G (**1**) [3], the new phenanthro-perylene quinone pigments gymnochrome H (**2**) and monosulfated gymnochrome A (**3**), as well as the known compounds gymnochrome A (**4**) and monosulfated gymnochrome D (**5**) (Figure 1).

**Figure 2:**
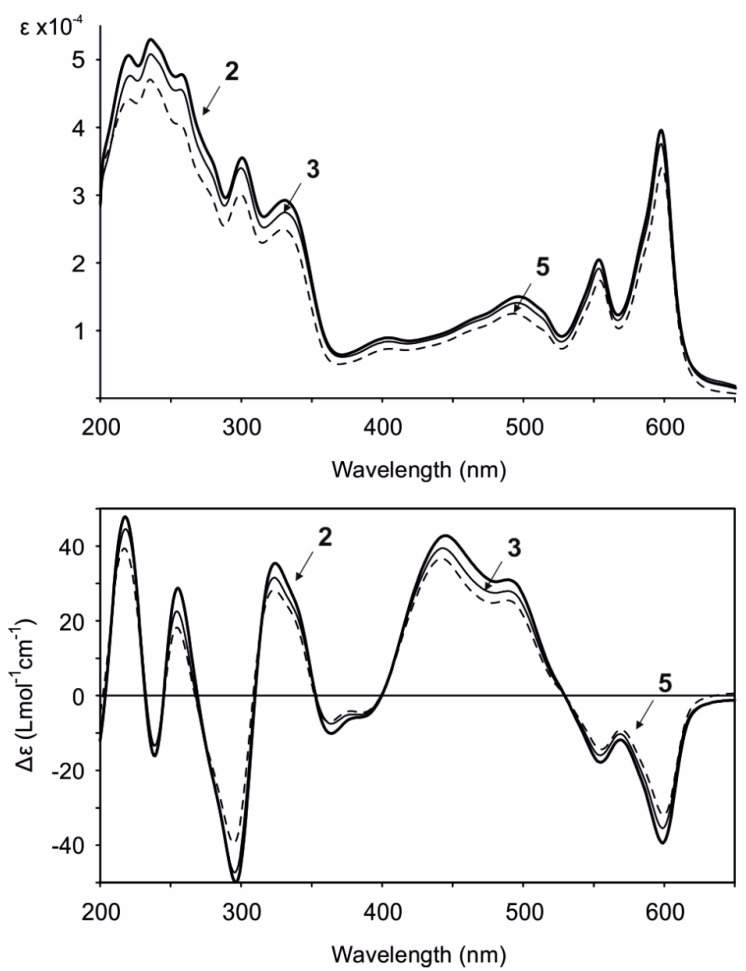
UV/vis (top) and ECD (bottom) spectra (MeOH) of gymnochrome H (**2**), monosulfated gymnochrome A (**3**), and monosulfated gymnochrome D (**5**).

**Figure 3:**
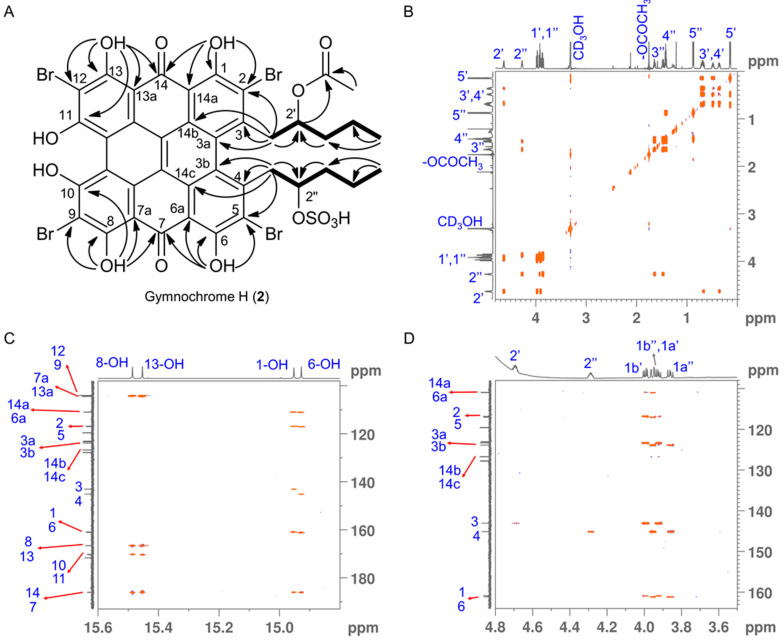
(**A**) ^1^H-^1^H COSY (bold lines) and key ^13^C-^1^H HMBC (H→C, OH→C and OH→CO) correlations for gymnochrome H (**2**). (**B**) Fully assigned ^1^H-^1^H COSY spectrum of gymnochrome H (**2**). (**C**,**D**) Selected regions of ^13^C-^1^H HMBC spectrum of gymnochrome H (**2**) recorded at 280 K highlighting the long-range carbon-proton (^2^*J*, ^3^*J*, and ^4^*J*) correlations that are crucial for the assignment of aromatic carbons. Proton and carbon assignments are shown on the 1D spectra.

Gymnochrome H (**2**) was isolated as a violet solid. It showed UV/vis absorption maxima (MeOH) at 220, 236, 258, 301, 331, 404, 496, 554, and 598 nm (Figure 2), very similar to those of phenanthroperylene quinones. The ESIMS spectrum showed a complex multiplet at *m/z* 1081, 1083, 1085, 1087, and 1089 (relative intensities 1.0:3.7:6.0:4.2:1.4) for the deprotonated molecular ion, indicating the presence of four bromine atoms. HRESIMS data of **2** suggested the molecular formula C_40_H_30_Br_4_O_14_S (*m/z* 1080.7993 [M – H]^–^). The characteristic fragment peak (monoisotopic) at *m/z* 983 [M – H_3_SO_4_]^–^ suggested the presence of a sulfate group. The ^1^H NMR spectrum of **2** (Table 1) showed no aromatic proton signals, indicating a highly condensed aromatic structure. The ^13^C APT NMR spectrum (Table 1) revealed the presence of 40 carbon signals: two carbonyl carbons at *δ*_C_ 185.9 and 185.8, 26 non-protonated aromatic carbons, including six phenolic carbons at *δ*_C_ 170.4, 170.3, 166.47, 166.46, 160.9, and 160.7 consistent with a substituted phenanthroperylene quinone structure, as well as one acetyl carbonyl carbon at *δ*_C_ 171.5 and 11 aliphatic carbons at *δ*_C_ 80.0 and 74.1 (methine groups), *δ*_C_ 44.2, 42.5, 38.8, 35.4, 18.9, and 18.1 (methylene groups), and *δ*_C_ 20.6, 14.4, and 13.4 (methyl groups). HMBC and COSY correlations indicated the presence of two pentyl side chains located in position 3 and 4 (Figure 3). The chemical shift of C2″ (*δ*_C_ 80.0) compared to that of C2′ (*δ*_C_ 74.1) indicated the presence of the sulfate group attached to C2″. An HMBC correlation from H2′ to 2′-O*C*OCH_3_ (*δ*_C_ 171.5) revealed the presence of an acetate group. Variable temperature ^1^H NMR spectra (Figure S9) of **2** acquired in MeOH-*d*_3_ showed broad resonances of *peri*-hydroxy protons at high temperature, while sharp signals with high intensity occurred at low temperature. Consequently, a further HMBC spectrum was recorded at 280 K, which allowed for the direct assignment of the *peri*-hydroxy groups 1-OH, 6-OH, 8-OH, and 13-OH by HMBC correlations from the corresponding hydroxy protons. The *bay*-hydroxy groups 10-OH and 11-OH gave no signal in the ^1^H NMR spectrum (likely because no hydrogen-bonding to a carbonyl group is possible at this positions), however could be indirectly assigned by weak ^4^*J* HMBC correlations from the nearby hydroxy protons 8-OH and 13-OH to the phenolic carbons C10 and C11, respectively. Although assignment of aromatic carbons is generally difficult in substituted phenanthroperylene quinones, the low temperature HMBC spectrum enabled the unambiguous assignment of the majority of aromatic carbons through long-range ^2^*J*, ^3^*J*, and ^4^*J* correlations either from hydroxy protons or from H1′ and H1″ to aromatic carbons (Table 1, Figure 3C). Only C10a, C10b, and the central carbons C14d, C14e, C14f, and C14g were assigned based on plausibility.

Monosulfated gymnochrome A (**3**) was found to have the molecular formula C_36_H_24_Br_4_O_13_S, as established by HRESIMS data (*m/z* 1010.7562 [M – H]^–^). The ^1^H NMR and ^13^C NMR signals of **3** (Table 1) were very similar to those of the previously described gymnochrome A (**4**) [5]. However, the ^1^H NMR spectrum of **3** showed almost identical signals for a 2″-sulfoxypentyl side chain as observed in **2**, suggesting that **3** is the monosulfated derivative of gymnochrome A (**4**).

Gymnochrome A (**4**) and monosulfated gymnochrome D (**5**), the latter being the most abundant phenanthroperylene quinone in *Hypalocrinus*, have previously been reported from the crinoid *Neogymnocrinus richeri* [5]. Spectroscopic data were in agreement with previously published values with exception of the ECD spectra that showed much higher Δ*ε* values (Figure 2) than previously reported. As compound **5** has only been partially characterized by NMR, we further confirmed the structure by ^13^C NMR and HMBC. It has been supposed that monosulfated gymnochrome D (**5**) (and monosulfated isogymnochrome D) were artifacts produced by partial hydrolysis of gymnochrome D (and isogymnochrome D) during extraction under slightly acidic conditions [5]. However, because extraction of *Hypalocrinus* was not performed under acidic conditions, it appears that monosulfated gymnochrome D (**5**) in fact may be an original natural product.

**Figure 4:**
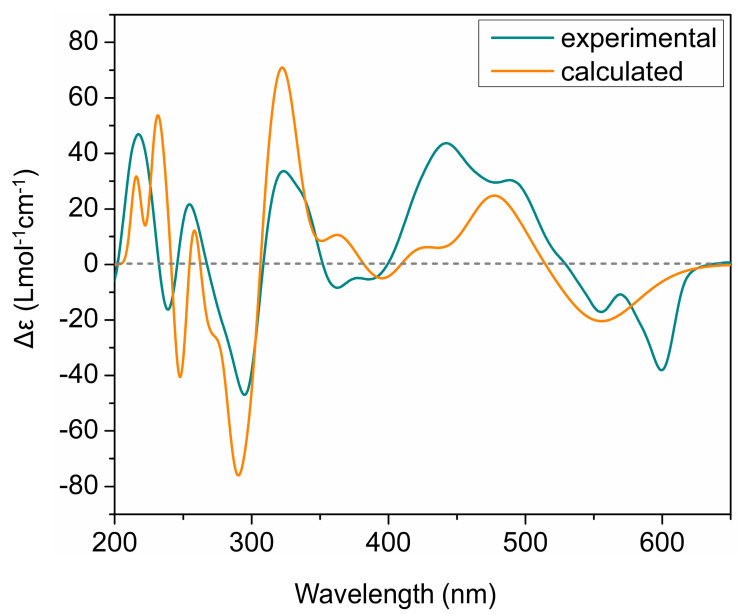
Comparison of experimental and calculated ECD curves of gymnochrome H (**2**) in MeOH. TDDFT ECD calculations were performed on low-energy conformations of **2** at PBE0/def2-tzvpp/def2tzv level of theory.

The ECD spectra of all isolated phenanthroperylene quinones (**2**–**5**) show a high similarity, independent from the length and configuration of the alkyl side chains (Figure 2). This strongly indicates that the ECD effect is dominated by the aromatic chromophore with only little influence from the alkyl side chains. Analysis of ECD spectra revealed that the configuration of the axial chirality of **2**–**5** is (*P*) (Figure 2). The propeller conformation of the gymnochromes with positive axial chirality was unambiguously confirmed by comparison of the measured ECD spectrum of gymnochrome H (**2**) with the calculated ECD curve of **2** (Figure 4) and those of the aromatic chromophore of the gymnochromes without alkyl side chains in propeller conformation with positive (*P*) and negative (*M*) axial chirality (Figure S10).

**Figure 5:**
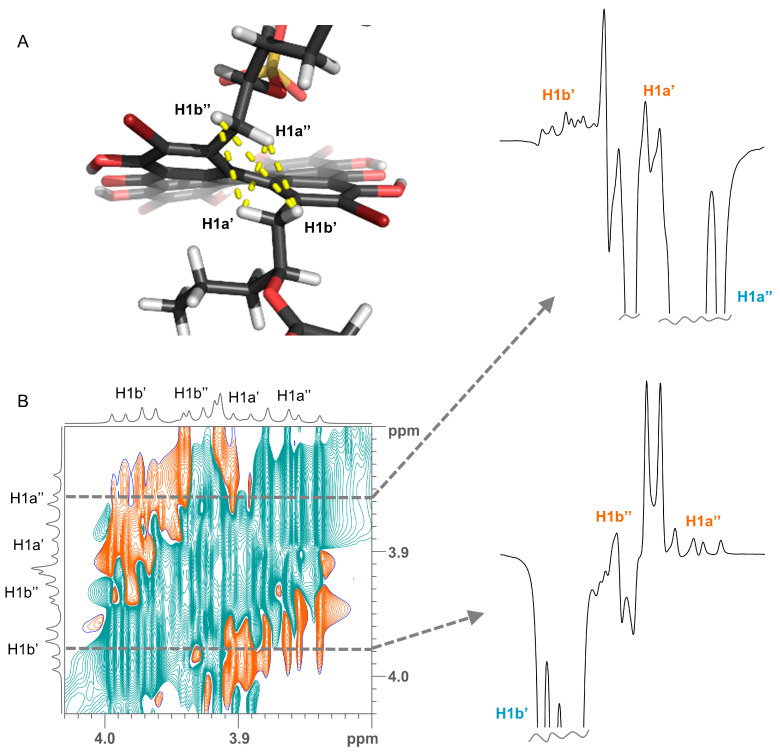
(**A**) Crucial ROE contacts for determining the orientation of side chains (yellow lines) shown on the example of a conformer of gymnochrome H (**2**). (**B**) A selected region of 2D ^1^H-^1^H ROESY spectrum of gymnochrome H (**2**). Slices extracted through the diagonal peaks of H1a″ and H1b’ (dashed lines) are shown on the right side. Proton assignments are denoted on the resonances.

**Figure 6:**
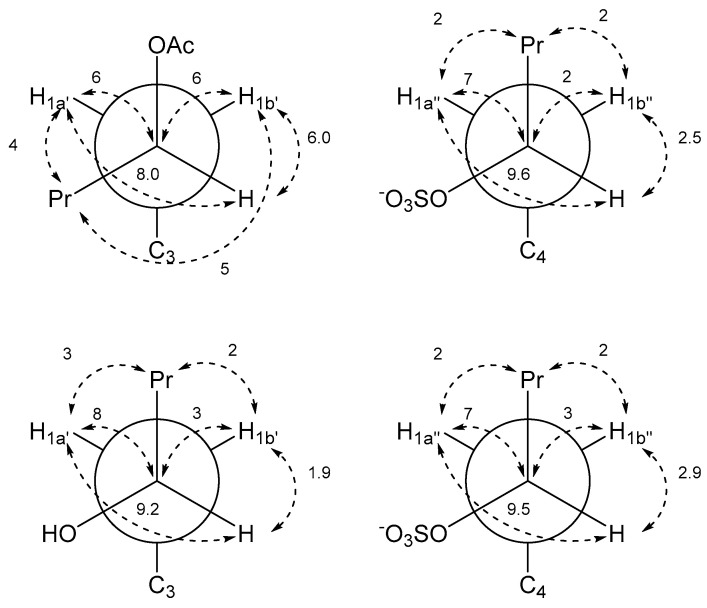
Newman projections of gymnochrome H (**2**) (top) and monosulfated gymnochrome D (**5**) (bottom) with measured *J*-couplings in Hz. Left projections are along C2′-C1′ and right projections are along C2″-C1″.

Determination of relative configuration of the side chains of **2** and **5** was done by determining the major staggered conformer from *J*-couplings as originally introduced by Pachler [9,10] and described for the natural product community 30 year later as Murata’s method [11]. The chain of arguments is very similar to the case of gymnochrome G (**1**) [3]. First, the only visible inter-side chain ROE contacts are between H1′ and H1″, which confirms the intuitive assumption that the side chains point away from each other and the chromophore (Figure 5). Second, the geminal protons H1a’/b’ and H1a″/b″ are assigned using *J*-couplings to C2/C5 and C3a/C3b (Table 2 and Table 3). Here, the relative coupling size can differentiate between syn- and antiperiplanar arrangements (antiperiplanar show a larger ^3^*J*_CH_-coupling). Finally, the couplings of the two protons H1′ to H2′, C2′, and C3′ (and H1″ to H2″, C2″, and C3″) are used: In all cases, H2′ (H2″) has a large and a small coupling to the two protons at position 1′ (1″), which indicate an antiperiplanar arrangement in the former and a synclinal arrangement in the latter case. The same connection between coupling size and arrangement applies to the coupling of C3′ (C3″) to the two protons H1′ (H1″), which allows us to deduce the arrangement of C3′ (C3″). Finally, the coupling of C2′ (C2″) to the protons indicates the dihedral angle between H1′ (H1″) and the electronegative substituent OR. However, here a large coupling indicates a synclinal arrangement and a small coupling an antiperiplanar arrangement. For both **2** and **5**, this yields the arrangement of the substituents at C2′ and C2″ and thus the configuration and dominant conformation. This is illustrated as a Newman projection in Figure 6, including the values of the couplings used in the deduction. These side chain configurations are relative to the configuration of the aromatic system. As the absolute configuration of the aromatic system is known to be (*P*), this therefore also establishes the absolute configurations of the side chains. The configuration is (2′*S*,2″*R*) for gymnochrome H (**2**), while it is (2′*R*,2″*R*) for monosulfated gymnochrome D (**5**) which is in agreement with previous results on **5** determined by De Riccardis et al. [5]. We corroborated these configurations using DP4+ [12,13], an approach based on Bayesian statistics that compares measured chemical shifts with those predicted by density functional theory (DFT). This confirmed the configurations determined via ROE and *J*-couplings with high confidence, and it allowed us to determine the configuration of the low-abundance monosulfated gymnochrome A (**3**) to be (2′*S*,2″*R*) (Table 4).

Our results further show that the proton chemical shifts in the propyl and pentyl side chains indeed are indicative of the configuration of gymnochromes as suggested by [5] and applied by [8]. In (*P*)-configured gymnochromes, the methyl group of propyl and the methyl groups of pentyl are in the range of 0 ppm for (*S*) configuration and in the range of 0.9 ppm in the case of (*R*) configuration, whereas in (*M*)-configured gymnochromes the methyl group of propyl and the methyl groups of pentyl are in the range of 0.9 ppm for (*S*) configuration and in the range of 0 ppm in the case of (*R*) configuration. This pattern also reaffirms the configuration of monosulfated gymnochrome A (**3**), which was only determined via DP4+. The propyl side chain methyl of **3** is shielded (*δ*_H_ −0.15) and therefore lies above the aromatic ring system. As axial chirality of **3** reveals (*P*) configuration, (*S*) configuration is suggested for the chiral center C2′. The smaller shielding (*δ*_H_ 0.88) of terminal methyl protons relative to the previously mentioned *δ*_H_ = −0.15 indicate that the pentyl side chain of **3** is directed away from the aromatic ring system, thus suggesting (*R*) configuration for the chiral center C2″.

It can be generally observed that in gymnochromes, independent from their axial chirality, chiral centers of propyl side chains have the (*S*) configuration, while chiral centers of pentyl side chains have the (*R*) configuration [3,5,8]. Possibly this is the result of their polyketide biosynthesis. This pattern can also be found in the gymnochromes described in this work, however, with the exception of gymnochrome H (**2**) whose 2-hydr-oxypentyl group is acetylated and is found in the (*S*) configuration.

Acetylated quinones are not common among natural products, especially among echinoderms. However, acetylated anthraquinones have recently been isolated from the crinoid *Pterometra venusta* [14]. Gymnochrome G (**1**) and H (**2**) represent the first acetylated phenanthroperylene quinone pigments. Previous HPLC-MS analysis of a crude extract of *Hypalocrinus*, also revealing the ion signals of **1** and **2** [2], proves that they are original natural products and no artifacts that may have formed during isolation of compounds.

As cytotoxic effects have been reported for gymnochrome E and F isolated from the crinoid *Holopus* [8], the cytotoxic activities of monosulfated gymnochrome D (**5**) were tested against the cell lines HT29, A549, MDA-MB-231, and PSN1, however, no inhibition at 25 μg/mL could be observed. Although the biological activities of the new gymnochromes have not been further investigated in this study, it is likely that they exhibit antiviral activity as observed for other gymnochromes and brominated hypericins. For example, gymnochrome D and isogymnochrome D were found to have activity against dengue virus [15,16], and gymnochrome B was found to have activity against herpes simplex virus and influenza virus [17].

Our results further confirm previous observations that many quinone pigments in crinoids occur as water-soluble sulfate esters [1,3,18,19,20], and the majority of quinones from stalked deep-sea crinoids are brominated in contrast to their relatives living in shallow water [19]. The diversity of pigments in the deep-sea crinoid *Hypalocrinus* is remarkable comprising highly substituted anthraquinones and biaryl quinones [1] as well as phenanthroperylene quinones, in total at least fifteen quinones with ten of them having never been observed before.

## 3. Materials and Methods

### 3.1. General Experimental Procedures

UV spectra were recorded on a Jasco V-630 UV–visible spectrophotometer, CD spectra were recorded on a Jasco J-810 spectropolarimeter, and IR spectra were measured on a Jasco 4100 FT-IR spectrometer equipped with a Pike Gladi ATR (attenuated total reflection) accessory. 1D and 2D NMR spectra were recorded in MeOH-*d*_3_ (with the exception of the known compound **4** that was recorded in MeOH-*d*_4_) at 298 K on Bruker Avance III HD spectrometers at 600, 800, and 900 MHz and an Avance Neo 800 MHz spectrometer, equipped with TCI (800/900) and QCI (600) probes. Measurement of NMR spectra with MeOH-*d*_3_ as the solvent facilitated the observation of *peri*-hydroxy (1-OH, 6-OH, 8-OH, and 13-OH) protons and thereby the assignment of aromatic carbons, which however is not possible using MeOH-*d*_4_. Chemical shifts were referenced using residual solvent peaks (MeOH: *δ*_H_ = 3.31 ppm, *δ*_C_ = 49.0 ppm). Standard pulse sequences for ^13^C–^1^H HSQC and ^13^C–^1^H HMBC experiments were used. The refocusing delays for the inverse heterocorrelations were set to 3.57 and 71.4 ms, corresponding to ^1^*J*_C,H_ = 140 Hz and *^n^J*_C,H_ = 7 Hz, respectively. For compound **2**, an additional HMBC (optimized for 2 Hz carbon-proton long-range coupling) spectrum was recorded at 280 K. The 2D ROESY spectrum of compound **2** was recorded using the Bruker standard pulse sequence (*roesyphpr.2*) with an alternating phase (180x 180-x) spin-lock pulses, a mixing time of 200 ms, and a spin-lock field strength of 2.8 kHz. A matrix of 8 k × 1 k complex points was used with 6600 Hz spectral width in both dimensions. A recovery delay of 2 s and 32 scans per increment were employed. *J*_HH_-couplings were determined from 1D ^1^H spectra. *^n^J*_CH_-couplings were determined using a *J*-HMBC [21] spectrum for compound **2** and a modified HMBC following the approach from Edden et al. [22] for compound **5**. High-resolution MS spectra were obtained using a Bruker micrOTOF mass spectrometer with electrospray ionization in the negative-ion mode. HPLC was performed on an Agilent 1200 Series system using a Phenomenex Gemini C18 column (250 × 10 mm i.d., 5 μm).

### 3.2. Animal Material

Two specimens of *H. naresianus* were collected in 2004 and 2008 by beam trawling from Shima Spur, Kumano-nada Sea, Japan from depths of 763 to 852 m. Voucher samples were deposited in the collection of the Systematische Zoologie am Museum für Naturkunde Berlin (ZMB Ech 7415 and ZMB Ech 7416).

### 3.3. Extraction and Isolation

Lyophilized *H. naresianus* material (9.0 and 10.1 g) was successively extracted with MeOH/CH_2_Cl_2_ (1:1), MeOH/H_2_O (9:1), MeOH/H_2_O (1:1), and distilled water. The methanol-soluble pigments of the MeOH/CH_2_Cl_2_ extract were combined with the other extracts and subjected to semipreparative HPLC using a linear gradient of acetonitrile/20 mM aqueous ammonium acetate (45:55) to 85% acetonitrile in 40 min at a flow rate of 4.5 mL/min. Those fractions containing compound **2** and **3** were further purified by semi-preparative HPLC using a linear gradient of MeOH/20 mM aqueous ammonium acetate (65:35) to 85% MeOH in 40 min at a flow rate of 4.5 mL/min. Fractions were concentrated using solid phase extraction (Bondesil C18, 40 µm). Pigments were washed with water and eluted with MeOH/H_2_O (9:1) followed by evaporation of the eluates to dryness to give the compounds **2** (2.1 mg), **3** (0.8 mg), **4** (0.4 mg), and **5** (4.1 mg).

**Gymnochrome H (2):** violet solid; UV (MeOH) *λ*_max_ (log *ε*) 220 (4.70), 236 (4.72), 258 (4.68), 301 (4.55), 331 (4.47), 404 (3.95), 496 (4.18), 554 (4.31), 598 (4.60); ECD (MeOH) *λ* (Δ*ε*) 218 (+47.86), 239 (−16.08), 255 (+28.80), 296 (−49.97), 324 (+35.40), 364 (−10.13), 445 (+42.80), 489 (+30.99), 555 (−17.83), 599 (–39.43); IR (ATR) *ν*_max_ 1569, 1449, 1236, 1128 cm^–1^; ^1^H NMR (MeOH-*d*_3_, 800 MHz) and ^13^C NMR (MeOH-*d*_3_, 200 MHz), see Table 1; HRESIMS *m/z* 1080.7993 [M − H]^–^ (calcd for C_40_H_29_Br_4_O_14_S, 1080.8017), 982.8343 [M − H_3_SO_4_]^–^ (calcd for C_40_H_27_Br_4_O_10_, 982.8343), 539.8985 [M − 2H]^2–^ (calcd for 0.5 · C_40_H_28_Br_4_O_14_S, 539.8972).

**Monosulfated gymnochrome A (3):** violet solid; UV (MeOH) *λ*_max_ (log *ε*) 221 (4.68), 236 (4.71), 257 (4.66), 300 (4.53), 331 (4.44), 404 (3.92), 495 (4.15), 554 (4.28), 598 (4.58); ECD (MeOH) *λ* (Δ*ε*) 218 (+44.59), 239 (−13.36), 254 (+22.52), 295 (−47.25), 324 (+31.57), 364 (−7.50), 443 (+39.47), 489 (+27.98), 555 (–15.95), 599 (–35.46); IR (ATR) *ν*_max_ 1572, 1451, 1240, 1128 cm^–1^; ^1^H NMR (MeOH-*d*_3_, 800 MHz) and ^13^C NMR (MeOH-*d*_3_, 200 MHz), see Table 1; HRESIMS *m/z* 1010.7562 [M − H]^–^ (calcd for C_36_H_23_Br_4_O_13_S, 1010.7598), 912.7894 [M − H_3_SO_4_]^–^ (calcd for C_36_H_21_Br_4_O_9_, 912.7925), 504.8765 [M − 2H]^2–^ (calcd for 0.5 · C_36_H_22_Br_4_O_13_S, 504.8763).

**Monosulfated gymnochrome D (5):** violet solid; UV (MeOH) *λ*_max_ (log *ε*) 220 (4.65), 235 (4.67), 299 (4.48), 330 (4.40), 405 (3.86), 493 (4.10), 554 (4.24), 598 (4.53); ECD (MeOH) *λ* (Δ*ε*) 217 (+39.38), 239 (−13.63) 255 (+18.19), 295 (−39.46), 323 (+28.27), 363 (−7.05), 442 (+36.67), 489 (+25.49), 555 (−14.36), 599 (–31.98); IR (ATR) *ν*_max_ 1572, 1452, 1239, 1128 cm^–1^; ^1^H NMR (MeOH-*d*_3_, 800 MHz) and ^13^C NMR (MeOH-*d*_3_, 200 MHz), see Table 1; HRESIMS *m/z* 1038.7889 [M − H]^–^ (calcd for C_38_H_27_Br_4_O_13_S, 1038.7911), 940.8193 [M − H_3_SO_4_]^–^ (calcd for C_38_H_25_Br_4_O_9_, 940.8238), 518.8913 [M − 2H]^2–^ (calcd for 0.5 · C_38_H_26_Br_4_O_13_S, 518.8919).

### 3.4. DP4+ Analysis

The starting geometries were built in Maestro 11.4 [23]. For all configurations, the quinoid carbonyl acted as H-bond acceptor for the 1-, 6-, 8-, and 13-OH; the 11-OH group acted as H-bond acceptor for 10-OH; and 12-Br acted as H-bond acceptor for 11-OH. In all following steps, this OH conformation was kept fixed to greatly reduce sampling complexity. The sulfate group was built in its deprotonated form. The aromatic ring was built in propeller conformation (*P*). All four combinations of configuration at the two stereogenic centers were generated.

The conformational search was performed with Macromodel 11.8 [24] using the MMFF forcefield [25] in vacuum. The method was a Monte Carlo torsional sampling [26] of all non-terminal rotatable sidechain bond with 100,000 steps, a minimization convergence of 1.0 kJ/mol/Å, and an energy threshold of 21 kJ/mol. The resulting conformers were subjected to a finer minimization with 0.001 kJ/mol/Å convergence, and structures with a maximum atom deviation below 0.5 Å were discarded as duplicates. The conformers were clustered using torsional RMSD to 50 clusters, and the lowest energy conformation of each cluster was accepted as member of the conformational ensemble. This ensured the feasibility of the following, computationally expensive procedure.

All conformers from the ensemble were geometry optimized at the B3LYP [27,28,29,30]/6-31G [31] level of theory with Gaussian09 [32], and all conformers below 8.4 kJ/mol of the minimum energy (zero-point and free energy corrected) were discarded. The shielding constants were calculated at the MPW1PW91 [33,34]/6-31+G(d,p) level of theory using GIAO [35] and an implicit solvent model. The resulting shieldings were then averaged assuming a Boltzmann distribution and the energies from the geometry optimization, and the DP4+ probability was calculated using these averaged shieldings from all sidechain H- and C-atoms and the ipso- and ortho-C-atoms of the aromatic system. Unassigned methylene protons were assigned to the better-fitting value for each configuration.

### 3.5. ECD Calculations

TDDFT ECD [36] calculations were performed on 13 low-energy conformers of gymnochrome H (**2**) with propeller-(*P)* and (2′*S*,2″*R*) configurations of aromatic core and alkyl side chains, respectively, at PBE0 [37,38,39] /def2-tzvpp [40,41] /def2tzv level of theory with 150 excited states. A polarizable continuum model (iefpcm [42], solvent: methanol) was employed for the ECD calculations. The extraction of ECD curve of each conformer followed by Boltzmann averaging were carried out using SpecDis v1.71 [43,44]. The entire Boltzmann weighted ECD curve is shifted by 15 nm so-called “UV-shift” to compare with the experimental ECD of gymnochrome H (**2**). TDDFT ECD calculations were also performed on propeller-(*P)* and propeller-(*M)* conformations of aromatic chromophore of gymnochromes without alkyl side chains by employing the above-mentioned procedure.

### 3.6. Biological Assays

The cytotoxic activities of compound **5** were tested against NSLC A-549 human lung carcinoma cells, MDA-MB-231 human breast adenocarcinoma cells, HT-29 human colorectal carcinoma cells, and PSN1 human pancreatic carcinoma cells. The assays were performed according to the procedure described in Skehan et al. [45].

## Figures and Tables

**Table 1:** NMR spectroscopic data for gymnochrome H (**2**), monosulfated gymnochrome A (**3**), and monosulfated gymnochrome D (**5**) ^a^.

	2		3		5	
Position	*δ*_C_, mult.	*δ*_H_, mult. (*J* in Hz)	*δ*_C_, mult.	*δ*_H_, mult. (*J* in Hz)	*δ*_C_, mult.	*δ*_H_, mult. (*J* in Hz)
1/6	160.7, C/160.9, C		160.8, C/160.9, C		160.75, C/160.73, C	
2/5	116.7, C/116.9, C		116.0, C/116.9, C		116.2, C/116.6, C	
3/4	142.9, C/144.9, C		144.1, C/145.4, C		146.1, C/144.6, C	
3a/3b	123.2, C/123.7, C		123.4, C/124.0, C		124.1, C/124.4, C	
6a/14a	110.84, C/110.75, C		110.7, C/110.6, C		111.0, C/110.6, C	
7/14	185.9, C/185.8, C		185.9, C/185.8, C		185.9, C/185.5, C	
7a/13a	104.2, C/104.3, C		104.19, C/104.24, C		104.1, C/104.0 C	
8/13	166.46, C/166.47, C		166.4, C/166.4, C		166.0, C/165.9, C	
9/12	103.81, C/103.80, C		103.82, C/103.80, C		103.6, C/103.3, C	
10/11	170.3, C/170.4, C		170.2, C/170.3, C		169.3, C/169.5, C	
10a/10b	119.54, C ^b^/119.48, C ^b^		119.44, C ^b^/119.43, C ^b^		118.8, C ^b^/118.8, C ^b^	
14b/14c	126.59, C/126.60, C		126.45, C/126.47, C		126.3, C/126.7, C	
14d/14g	127.7, C ^b^/127.6, C ^b^		127.7, C ^b^/127.6, C ^b^		127.5, C ^b^/127.4, C ^b^	
14e/14f	123.0, C ^b^/122.9, C ^b^		122.94, C ^b^/122.91, C ^b^		123.1, C ^b^/122.9, C ^b^	
1′	42.5 CH_2_	3.91 dd (13.6; 7.9), 3.98 dd (13.7; 6.1)	48.8, CH_2_	3.62 dd (12.9; 9.8), 4.06 dd (12.8; 3.9)	48.3, CH_2_	3.68 dd (13.9; 9.0), 3.93 dd (13.9; 1.3)
2′	74.1, CH	4.63 m	68.2, CH	3.69 m	73.1, CH	3.75 m
2′-O*C*OCH_3_	171.5, C					
2′-OCO*CH_3_*	20.6, CH_3_	1.75 s				
3′	35.4, CH_2_	0.36 m, 0.68 m	21.1, CH_3_	–0.15 d (6.0)	41.2, CH_2_	1.47 m, 1.56 m
4′	18.1, CH_2_	0.48 m, 0.71 m			19.9, CH_2_	1.36 m, 1.43 m
5′	13.4, CH_3_	0.14 t (7.2)			14.2, CH_3_	0.90 t (7.2)
1″	44.2, CH_2_	3.86 dd (14.1; 9.6), 3.93 dd (14.2; 2.4)	44.1, CH_2_	3.87 m, 3.92 m	44.1, CH_2_	3.84 m, 3.89 m
2″	80.0, CH	4.27 m	79.9, CH	4.30 m	80.0, CH	4.27 m
3″	38.8, CH_2_	1.47 m, 1.64 m	38.8, CH_2_	1.49 m, 1.65 m	38.6, CH_2_	1.39 m, 1.59 m
4″	18.9, CH_2_	1.42 m	18.9, CH_2_	1.43 m	18.8, CH_2_	1.38 m
5″	14.4, CH_3_	0.87 t (7.3)	14.4, CH_3_	0.88 t (7.3)	14.4, CH_3_	0.83 t (7.1)
1-OH		14.92 s		14.92 s		14.88 s
6-OH		14.90 s		14.91 s		14.93 s
8-OH		15.48 s		15.49 s		15.47 s
13-OH		15.43 s		15.46 s		15.40 s

^a^ Spectra were recorded in MeOH-*d*_3_ at 800 MHz for ^1^H NMR and 200 MHz for ^13^C NMR. ^b^ Most plausible assignments are given, since no correlations to protons were found for these signals.

**Table 2:** ^13^C-^1^H and ^1^H-^1^H coupling constants for gymnochrome H (**2**).

Pentyl Acetate Side Chain		Pentyl Sulfate Side Chain	
*J*-HMBC Correlation	*J* in Hz ^a^	*J*-HMBC Correlation	*J* in Hz ^a^
^3^ *J*_C3a–H1a′_	5	^3^ *J*_C3b–H1a″_	4
^3^ *J*_C3a–H1b′_	5	^3^ *J*_C3b–H1b″_	5
^3^ *J*_C2–H1a′_	8	^3^ *J*_C5–H1a″_	8
^3^ *J*_C2–H1b′_	5	^3^ *J*_C5–H1b″_	5
^3^ *J*_H2′–H1a′_	8.0	^3^ *J*_H2″–H1a″_	9.6
^3^ *J*_H2′–H1b′_	6.0	^3^ *J*_H2″–H1b″_	2.5
^3^ *J*_C3–H2′_	2	^3^ *J*_C4–H2″_	
^3^ *J*_C3′–H1a′_	4	^3^ *J*_C3″–H1a″_	2
^3^ *J*_C3′–H1b′_	5	^3^ *J*_C3″–H1b″_	2
^2^ *J*_C2′–H1a′_	6	^2^ *J*_C2″–H1a″_	7
^2^ *J*_C2′–H1b′_	6	^2^ *J*_C2″–H1b″_	2

^a^ Coupling constants are absolute values without sign. The differences in significant digits in the coupling constants are due to the lower measurement accuracies of HMBC-based approaches compared to the extraction from 1D ^1^H NMR spectra.

**Table 3:** ^13^C-^1^H and ^1^H-^1^H coupling constants for monosulfated gymnochrome D (**5**).

Hydroxypentyl Side Chain		Pentyl Sulfate Side Chain	
*J*-HMBC Correlation	*J* in Hz ^a^	*J*-HMBC Correlation	*J* in Hz ^a^
^3^ *J*_C3a–H1a′_	4	^3^ *J*_C3b–H1a″_	4
^3^ *J*_C3a–H1b′_	5	^3^ *J*_C3b–H1b″_	5
^3^ *J*_C2–H1a′_	8	^3^ *J*_C5–H1a″_	8
^3^ *J*_C2–H1b′_	4	^3^ *J*_C5–H1b″_	4
^3^ *J*_H2′–H1a′_	9.2	^3^ *J*_H2″–H1a″_	9.5
^3^ *J*_H2′–H1b′_	1.9	^3^ *J*_H2″–H1b″_	2.9
^3^ *J*_C3–H2′_		^3^ *J*_C4–H2″_	
^3^ *J*_C3′–H1a′_	3	^3^ *J*_C3″–H1a″_	2
^3^ *J*_C3′–H1b′_	2	^3^ *J*_C3″–H1b″_	2
^2^ *J*_C2′–H1a′_	8	^2^ *J*_C2″–H1a__″_	7
^2^ *J*_C2′–H1b′_	3	^2^ *J*_C2″–H1b″_	3

^a^ Coupling constants are absolute values without sign. The differences in significant digits in the coupling constants are due to the lower measurement accuracies of HMBC-based approaches compared to the extraction from 1D ^1^H NMR spectra.

**Table 4:** DP4+ probabilities in percent for compounds **2**, **3**, and **5**.

	(2′*R*,2″*R*)	(2′*R*,2″*S*)	(2′*S*,2″*R*)	(2′*S*,2″*S*)
Gymnochrome H (**2**)	0.00	0.00	97.36	2.64
Monosulfated gymnochrome A (**3**)	0.00	0.00	100.00	0.00
Monosulfated gymnochrome D (**5**)	99.99	0.01	0.00	0.00

## Data Availability

Data are contained within the article or Supplementary Material.

## Supplementary Materials

The following are available online at https://www.mdpi.com/article/10.3390/md19080445/s1, Figures S1–S23: HRESIMS and NMR spectra of **2**, **3**, and **5**.
