# Supplementary material for: Structure and Absolute Configuration of Phenanthro-perylene Quinone Pigments from the Deep-Sea Crinoid Hypalocrinus naresianus
Source: Mar Drugs. 2021 Aug 3;19(8):445. doi: 10.3390/md19080445 (PMC8400451; doi:10.3390/md19080445)
Supplement: Supplementary file 1 [file marinedrugs-19-00445-s001.zip › marinedrugs-1314068-supplementary.pdf]

marine drugs

Supporting Information

Structure and Absolute Configuration of Phenanthroperylene Quinone Pigments from the Deep-Sea Crinoid *Hypalocrinus naresianus*

Sahithya Phani Babu Vemulapalli[1,2,†], Juan Carlos Fuentes-Monteverde[1,†], Niels Karschin[1,†], Tatsuo Oji[3], Christian Griesinger[1,*] and Klaus Wolkenstein[1,4,*]

[1] Department of NMR-Based Structural Biology, Max Planck Institute for Biophysical Chemistry, Am Fassberg 11, 37077 Göttingen, Germany

[2] Institute for Chemistry and Biology of the Marine Environment, University of Oldenburg, Carl-von-Ossietzky-Str. 9-11, 26129 Oldenburg, Germany

[3] Nagoya University Museum, Nagoya University, Nagoya 464-8601, Japan

[4] Department of Geobiology, Geoscience Centre, University of Göttingen, Goldschmidtstrasse 3, 37077 Göttingen, Germany

* Correspondence: klaus.wolkenstein@uni-goettingen.de (K.W.); cigr@nmr.mpibpc.mpg.de (C.G.)

† These authors contributed equally.

Table of Contents

Figure S1. HRESIMS spectrum (negative-ion mode) of gymnochrome H (**2**).
Figure S2. ^{1}H NMR spectrum (800 MHz, MeOH-d_3) of gymnochrome H (**2**).
Figure S3. ^{13}C APT NMR spectrum (200 MHz, MeOH-d_3) of gymnochrome H (**2**).
Figure S4. COSY spectrum of gymnochrome H (**2**).
Figure S5. ROESY spectrum of gymnochrome H (**2**).
Figure S6. HSQC spectrum of gymnochrome H (**2**).
Figure S7. HMBC spectrum of gymnochrome H (**2**).
Figure S8. *J*-HMBC spectrum of gymnochrome H (**2**).
Figure S9. Variable temperature ^{1}H NMR spectra of gymnochrome H (**2**).
Figure S10. ECD analysis of gymnochrome H (**2**).
Figure S11. HRESIMS spectrum (negative-ion mode) of monosulfated gymnochrome A (**3**).
Figure S12. ^{1}H NMR spectrum (800 MHz, MeOH-d_3) of monosulfated gymnochrome A (**3**).
Figure S13. ^{13}C APT NMR spectrum (200 MHz, MeOH-d_3) of monosulfated gymnochrome A (**3**).
Figure S14. COSY spectrum of monosulfated gymnochrome A (**3**).
Figure S15. HSQC spectrum of monosulfated gymnochrome A (**3**).
Figure S16. HMBC spectrum of monosulfated gymnochrome A (**3**).
Figure S17. HRESIMS spectrum (negative-ion mode) of monosulfated gymnochrome D (**5**).
Figure S18. ^{1}H NMR spectrum (800 MHz, MeOH-d_3) of monosulfated gymnochrome D (**5**).
Figure S19. ^{13}C APT NMR spectrum (200 MHz, MeOH-d_3) of monosulfated gymnochrome D (**5**).
Figure S20. COSY spectrum of monosulfated gymnochrome D (**5**).
Figure S21. HSQC spectrum of monosulfated gymnochrome D (**5**).
Figure S22. HMBC spectrum of monosulfated gymnochrome D (**5**).
Figure S23. *J*-HMBC spectrum of monosulfated gymnochrome D (**5**).

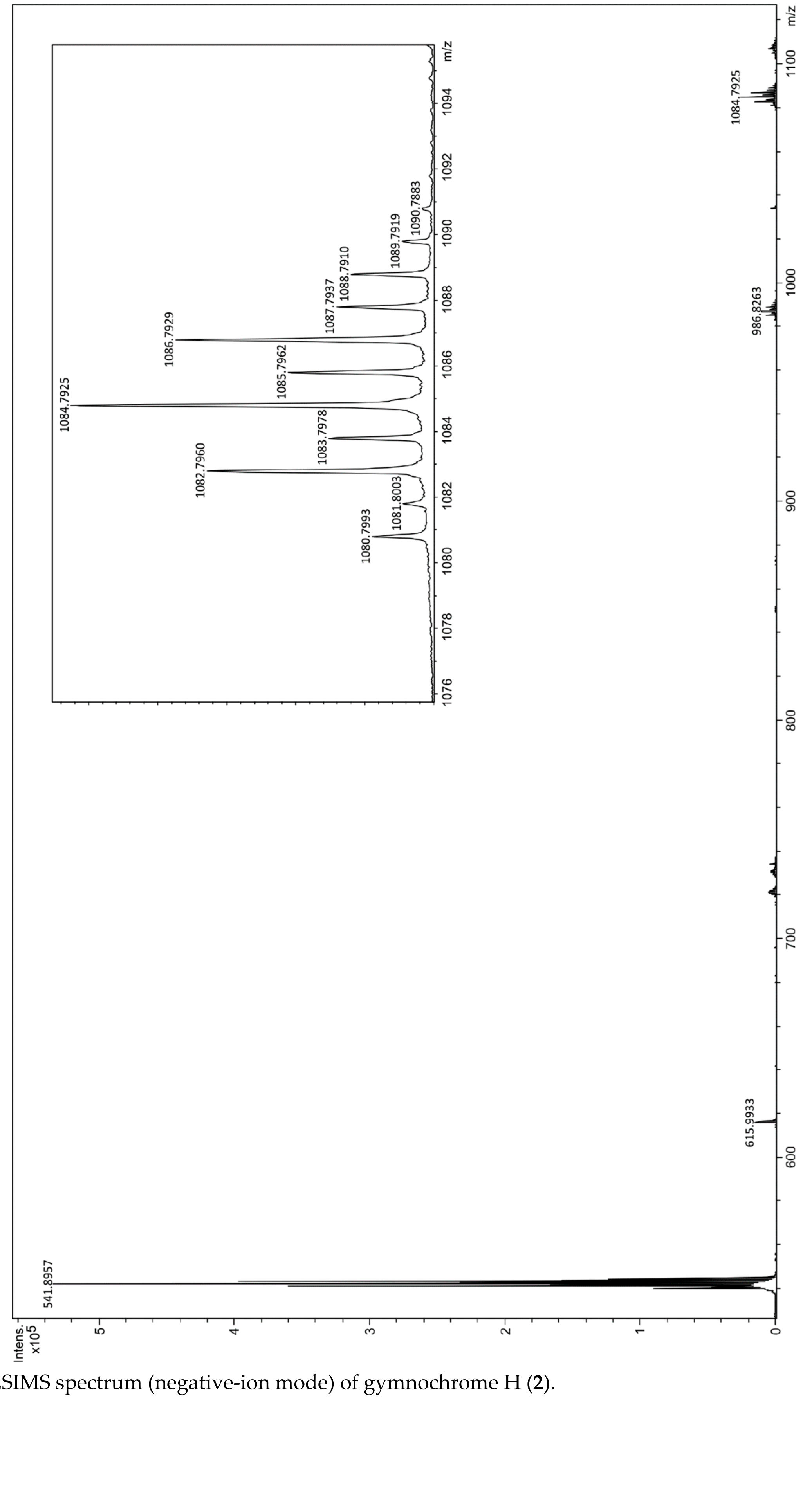

Figure S1. HRESIMS spectrum (negative-ion mode) of gymnochrome H (**2**).

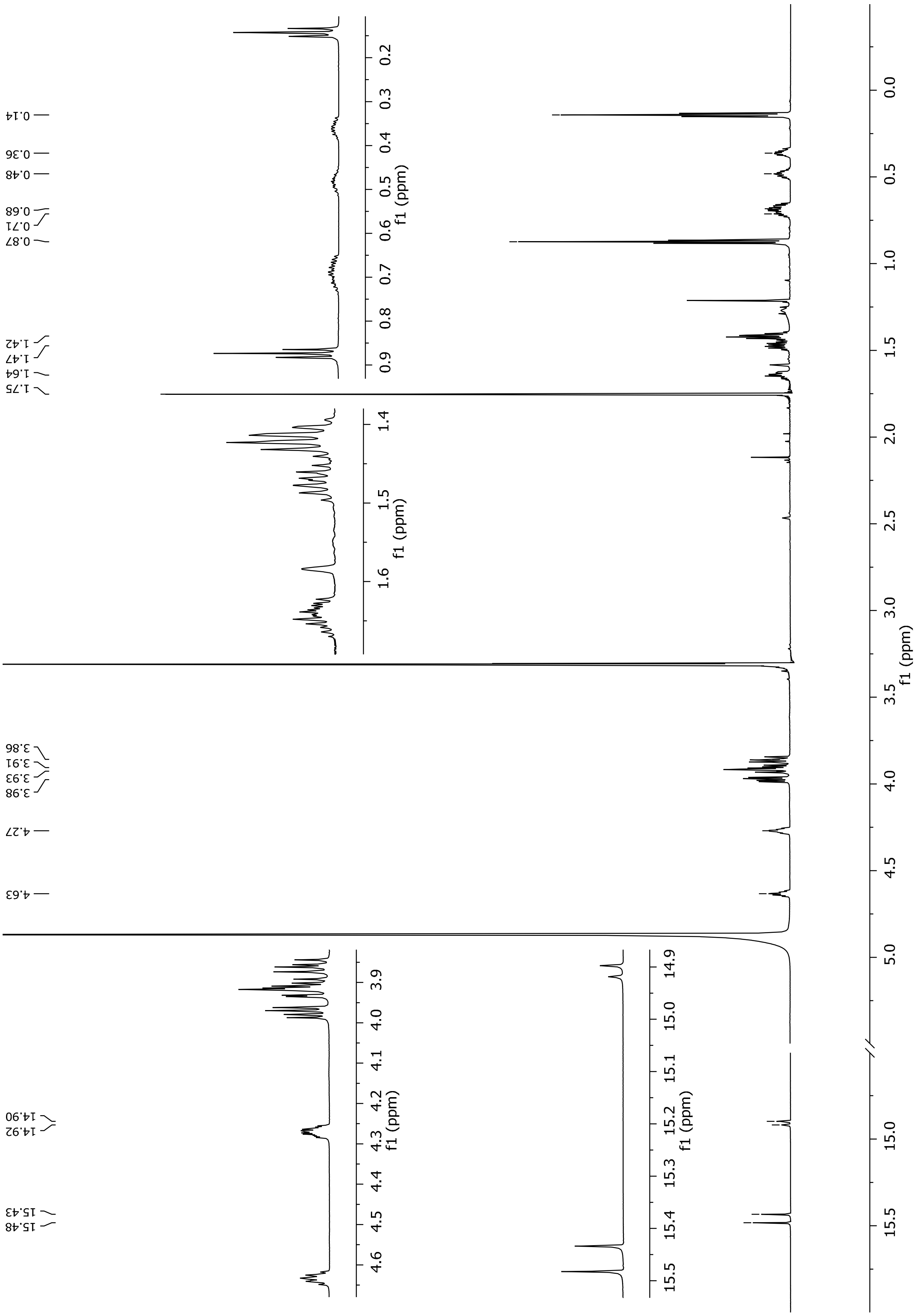

Figure S2. ^{1}H NMR spectrum (800 MHz, MeOH-d_3) of gymnochrome H (**2**).

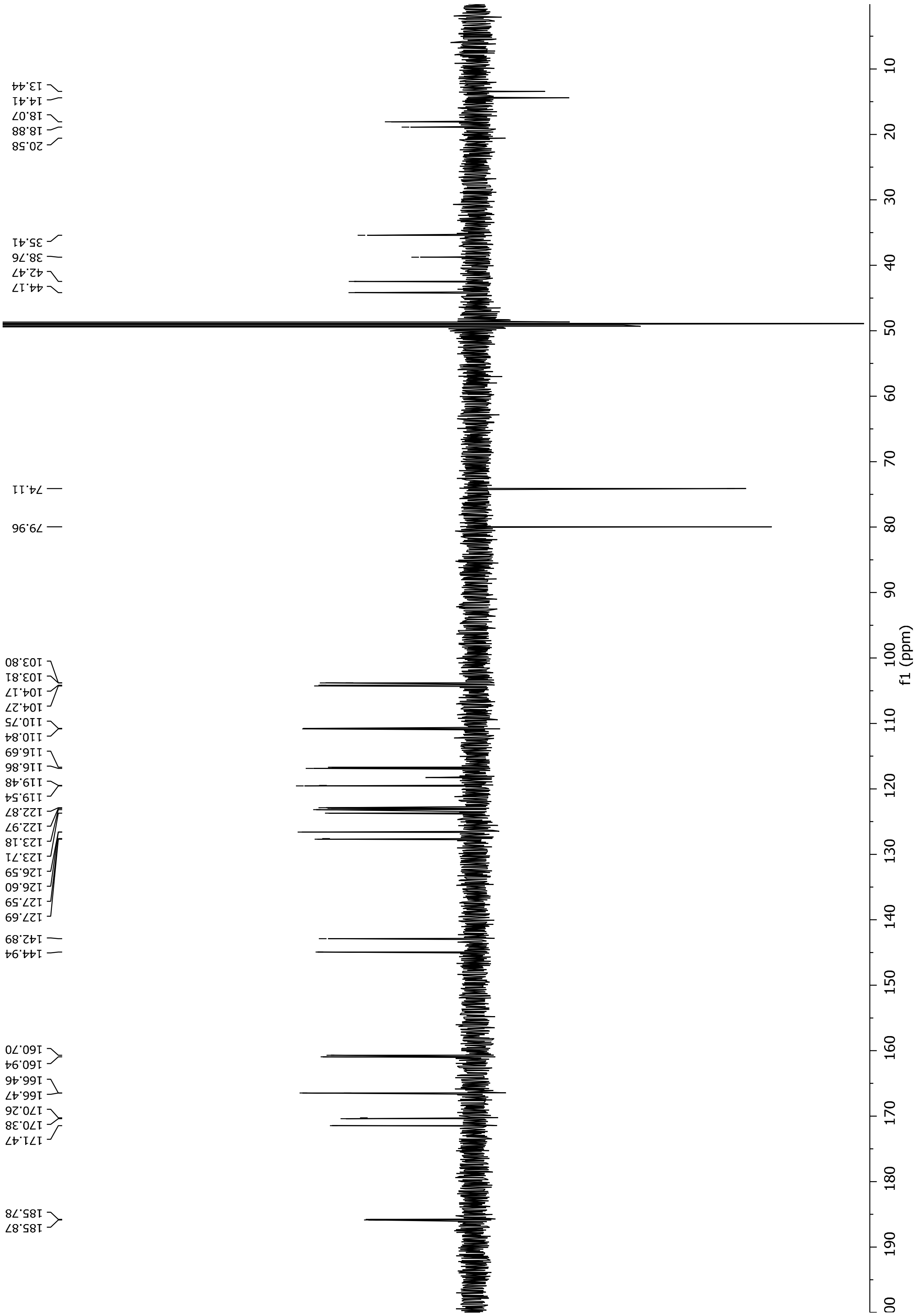

Figure S3. ^{13}C APT NMR spectrum (200 MHz, MeOH-d_3) of gymnochrome H (**2**).

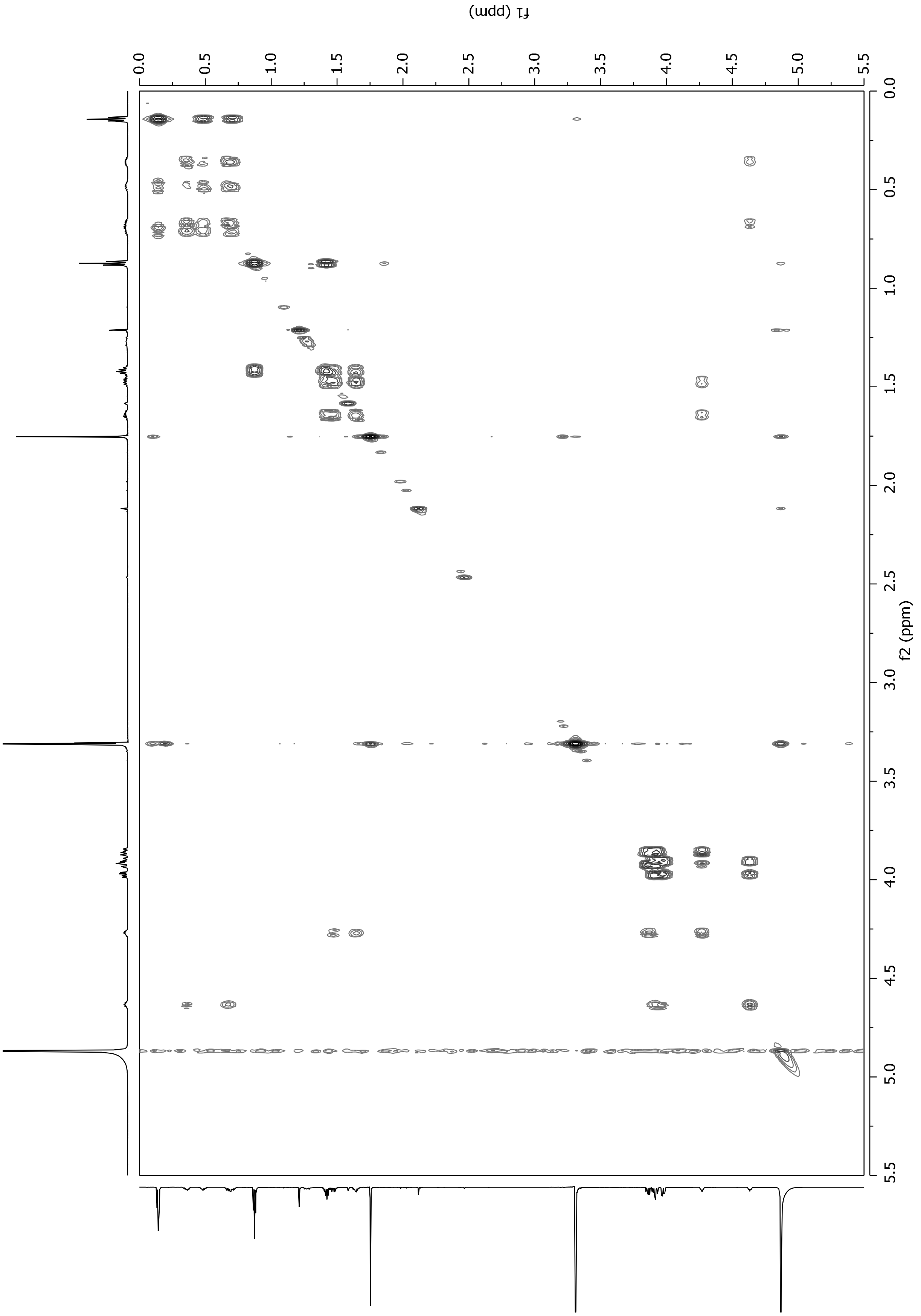

Figure S4. COSY spectrum (800 MHz, MeOH-d_3) of gymnochrome H (**2**).

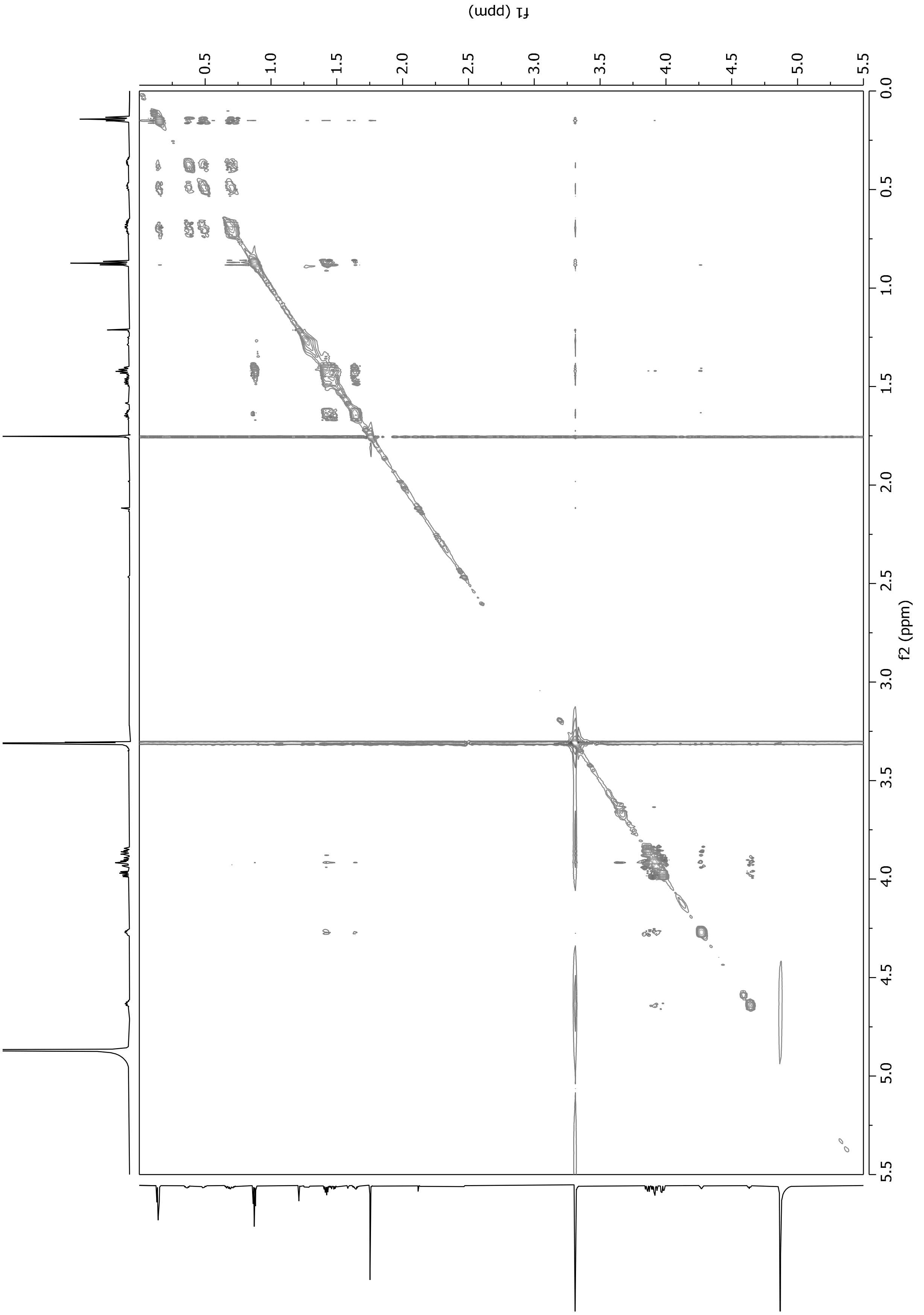

Figure S5. ROESY spectrum (600 MHz, MeOH-*d*3) of gymnochrome H (**2**).

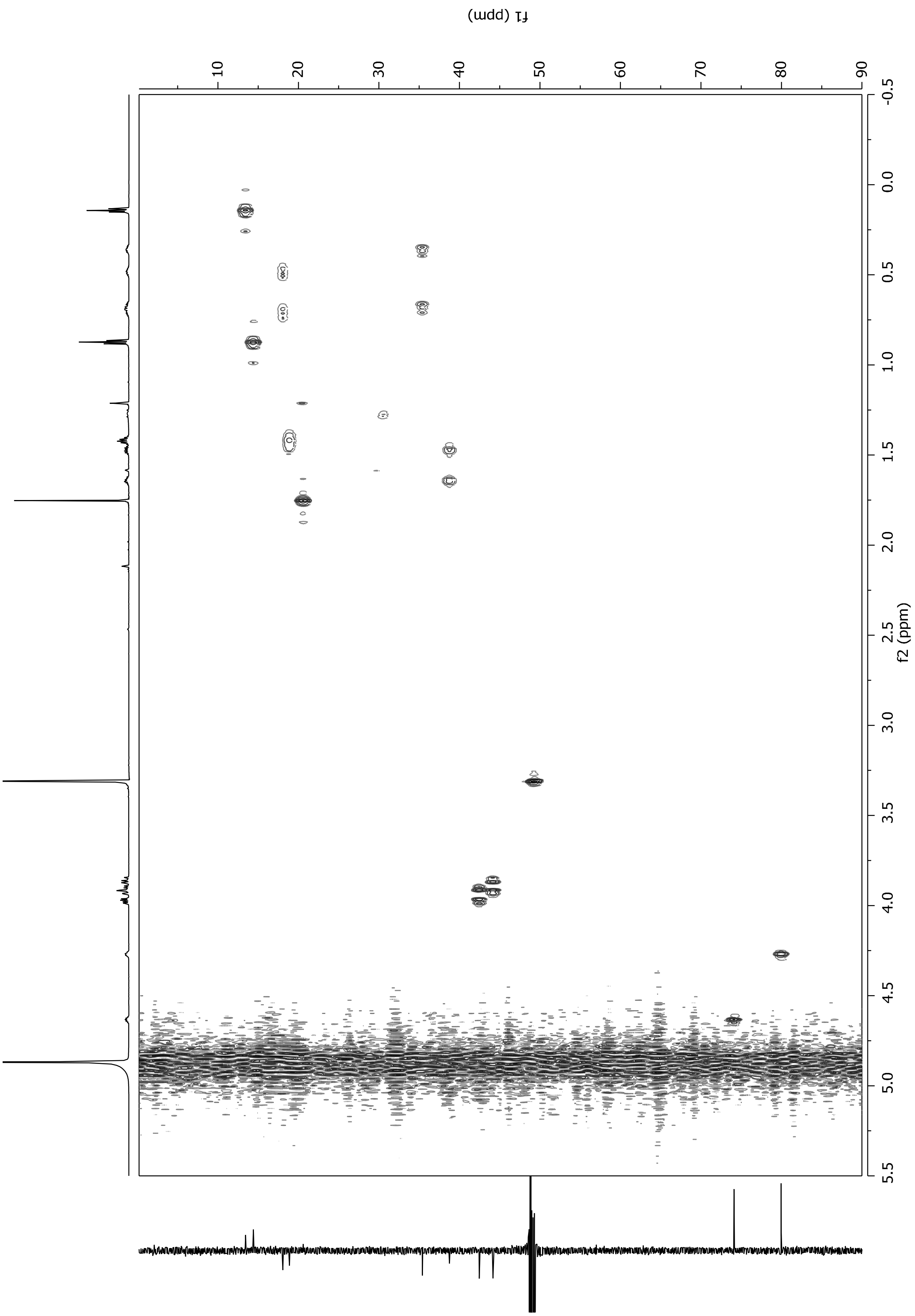

Figure S6. HSQC spectrum (800 MHz, MeOH-d_3) of gymnochrome H (**2**).

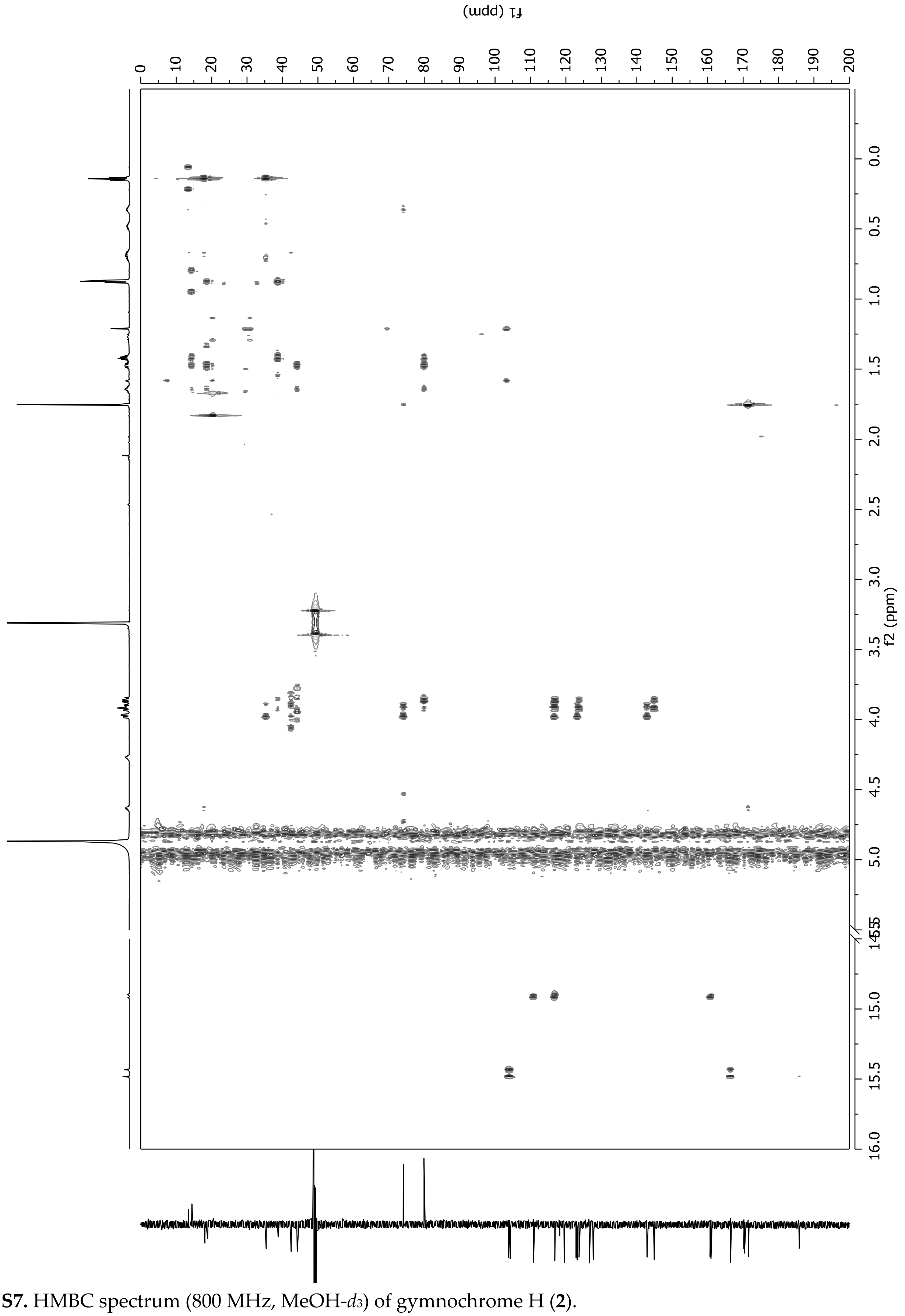

Figure S7. HMBC spectrum (800 MHz, MeOH-*d*3) of gymnochrome H (**2**).

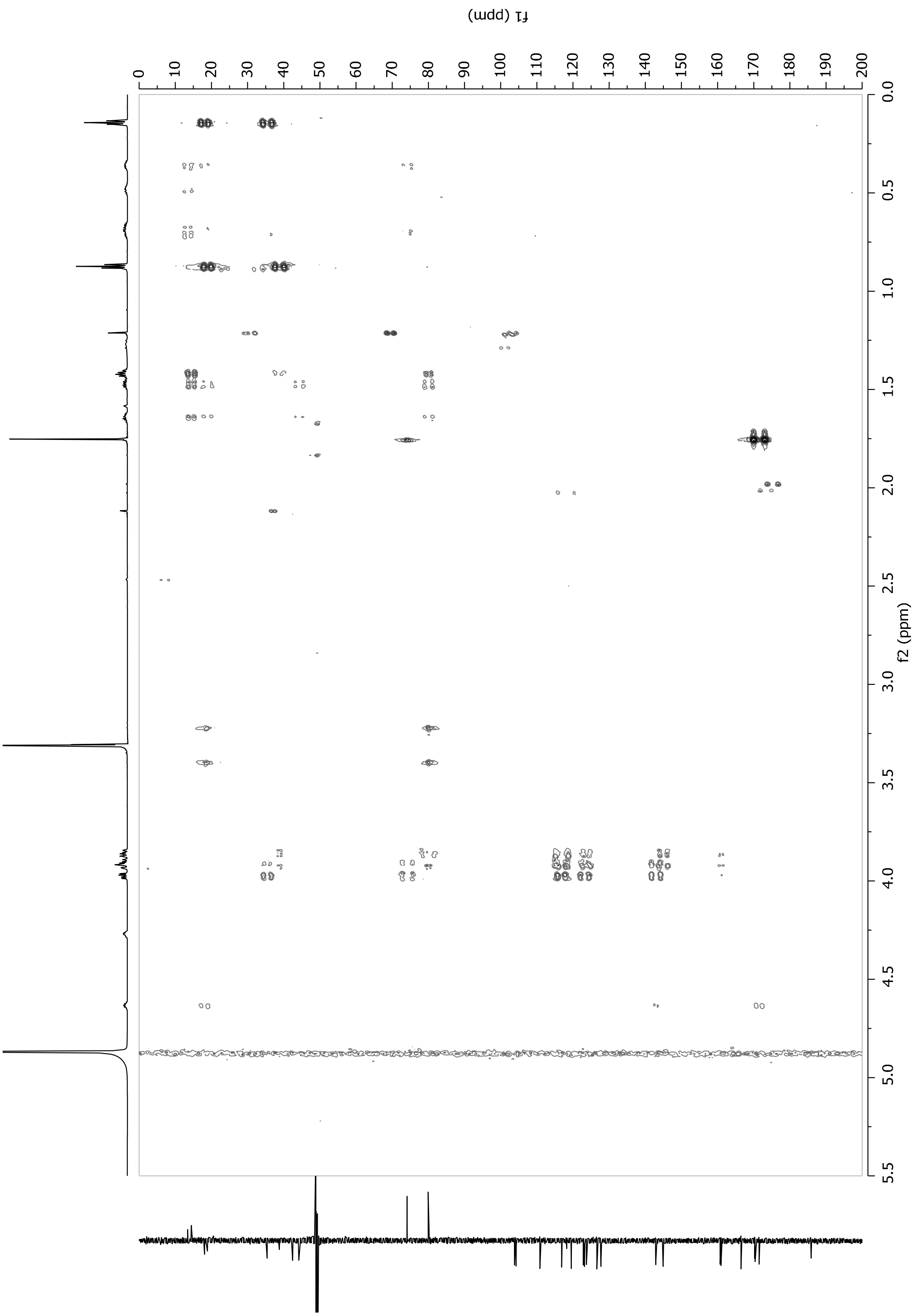

Figure S8. *J*-HMBC spectrum (800 MHz, MeOH-d_3) of gymnochrome H (**2**).

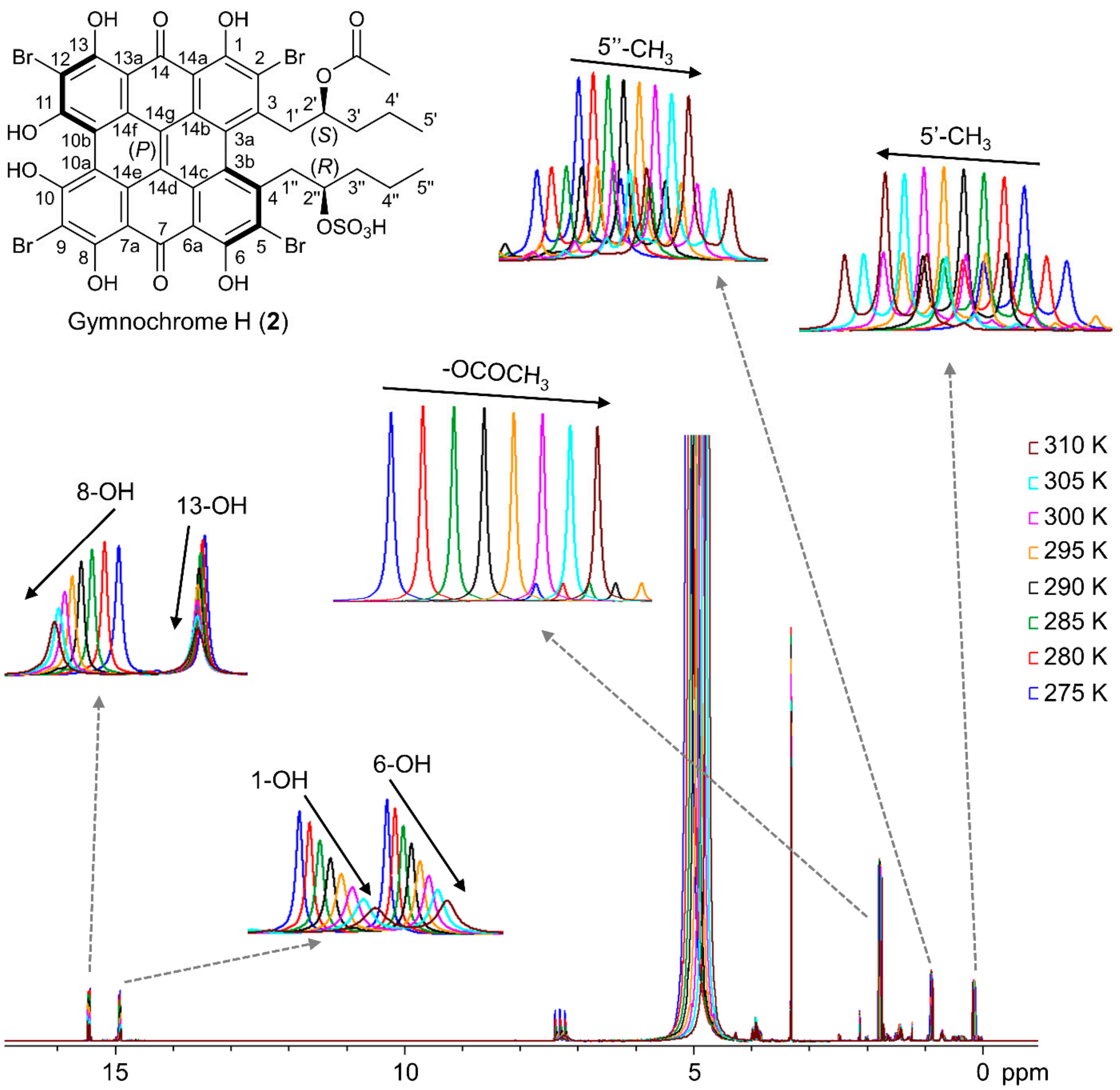

Figure S9. Variable temperature ^{1}H NMR spectra of gymnochrome H (**2**) acquired on Bruker Avance III HD 600 MHz spectrometer equipped with a 5 mm QCI cryo probe. Before each measurement, the sample was allowed to reach its thermal equilibrium for 15 min. 8-OH proton shifted downfield and 1-OH and 6-OH protons shifted upfield as the temperature increased from 275 to 310 K, which suggest further hydrogen bond strengthening and weakening for 8-OH and 1-OH/6-OH protons, respectively. While 13-OH proton marginally moved downfield. Terminal methyl protons 5′-CH_3 and 5′′-CH_3 shifted downfield and upfield, respectively, indicating the change in the orientation of the side chains there by change in the deshielding and shielding effects of aromatic ring system. Acetyl methyl protons are further shielded as the temperature increased from 275 to 310 K. Chemical structure of gymnochrome H (**2**) with atom numbering is shown above. Color of the each ^{1}H NMR spectrum is labeled with the respective temperature on the right side.

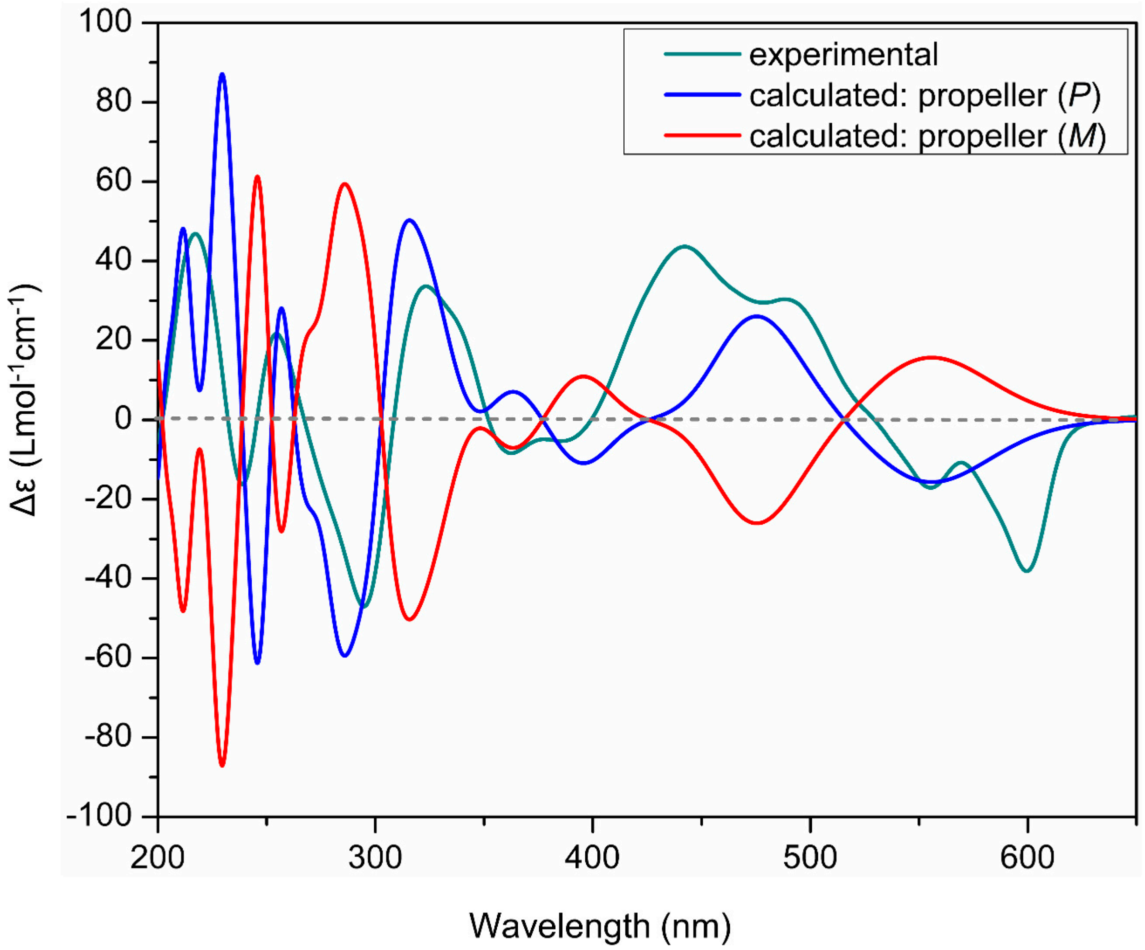

Figure S10. Blue and red lines are the calculated ECD curves of aromatic chromophore (without alkyl side chains) of gymnochromes in propeller conformation with (*P*) and (*M*) axial chirality, respectively. Experimental ECD curve (dark cyan) of gymnochrome H (**2**) is shown for reference. The comparison of experimental and calculated ECD curves strongly suggest the propeller conformation of aromatic chromophore of gymnochromes with (*P*) axial chirality, while propeller with (*M*) axial chirality produced enantiomeric ECD compared to experimental. It further highlights the fact that the ECD of gymnochromes is dominated by the aromatic chromophore with no influence from the alkyl side chains, which nicely correlates with the observed similar ECD curves of different gymnochromes.

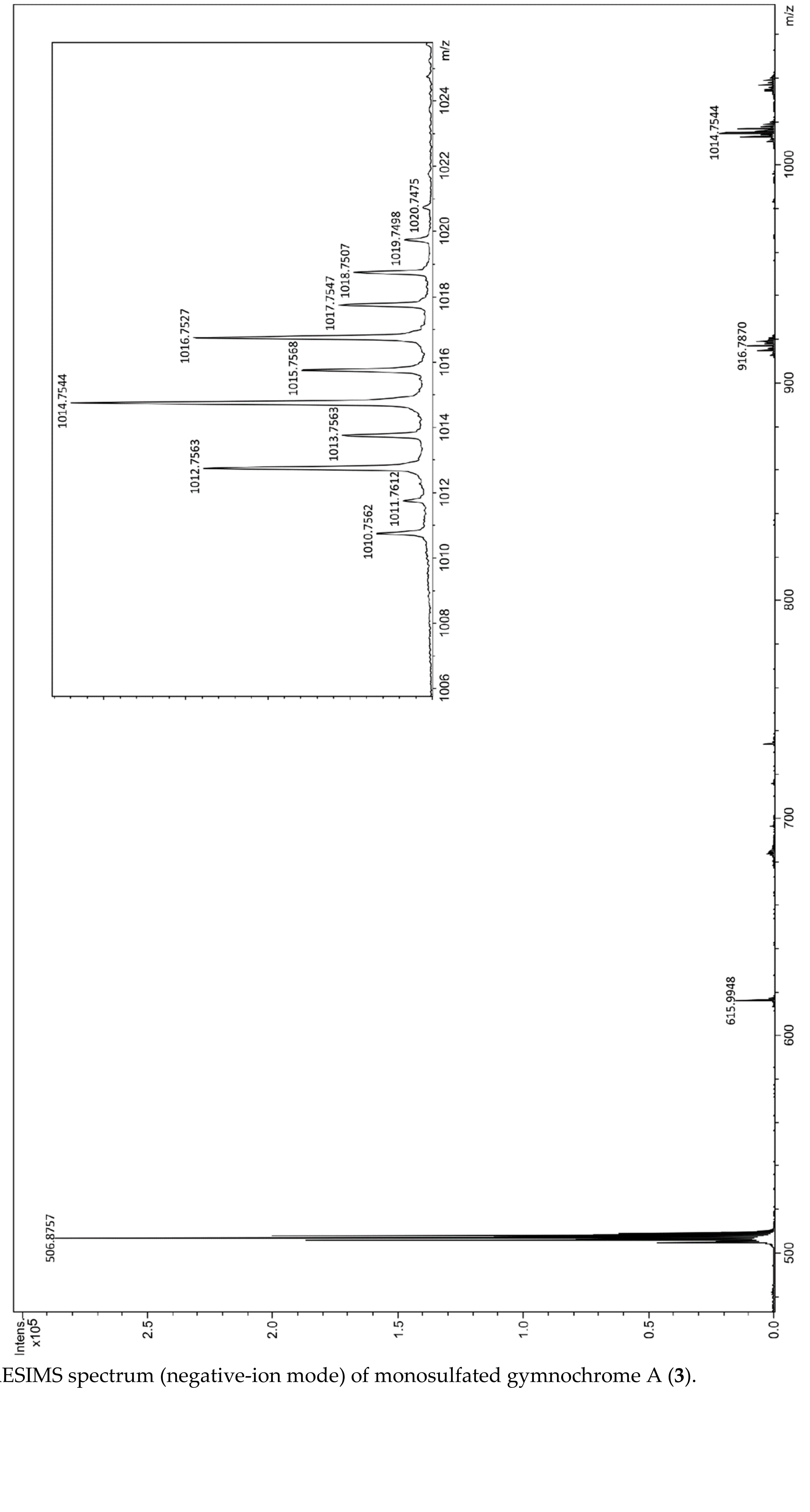

Figure S11. HRESIMS spectrum (negative-ion mode) of monosulfated gymnochrome A (**3**).

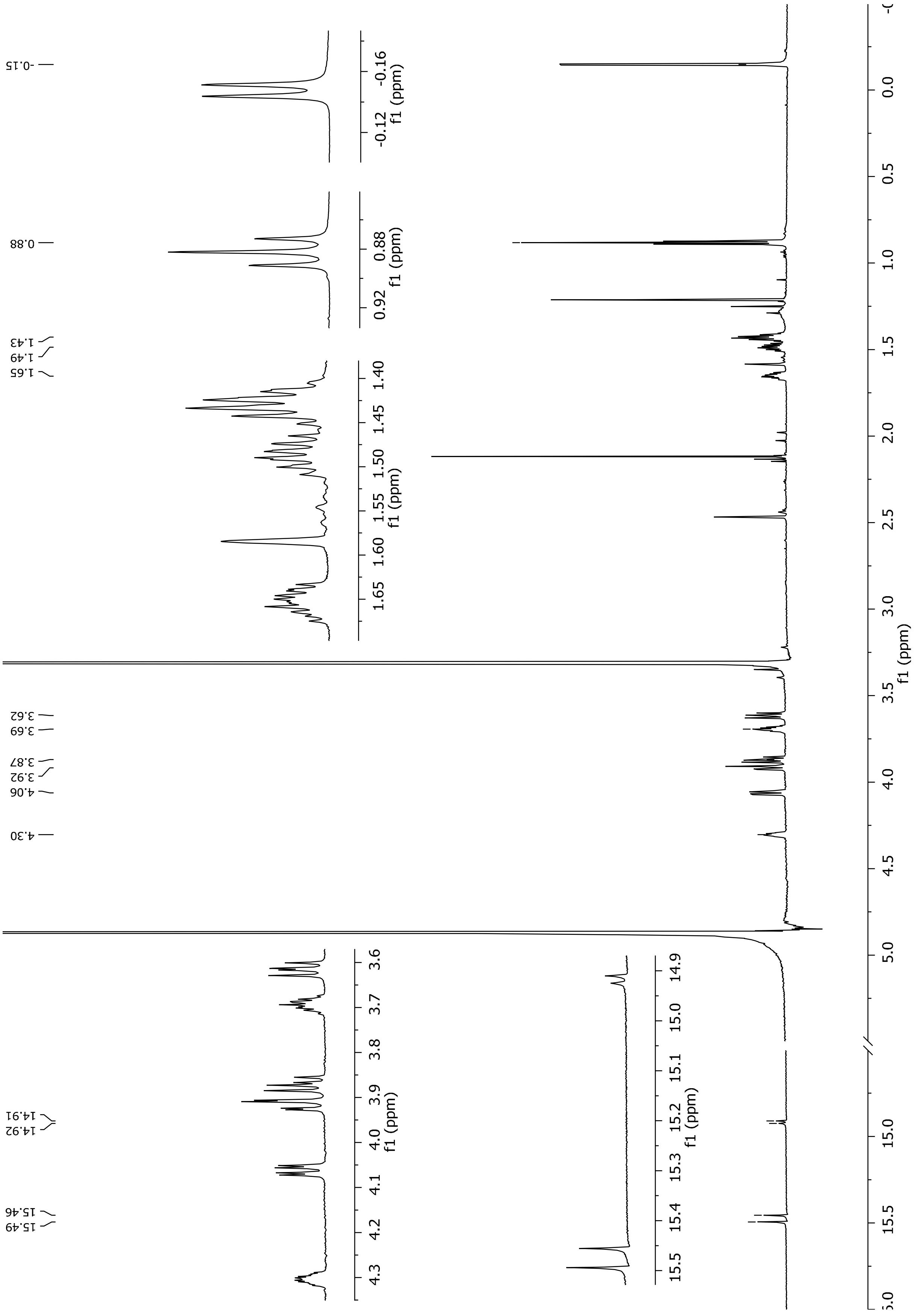

Figure S12. ^{1}H NMR spectrum (800 MHz, MeOH-d_3) of monosulfated gymnochrome A (**3**).

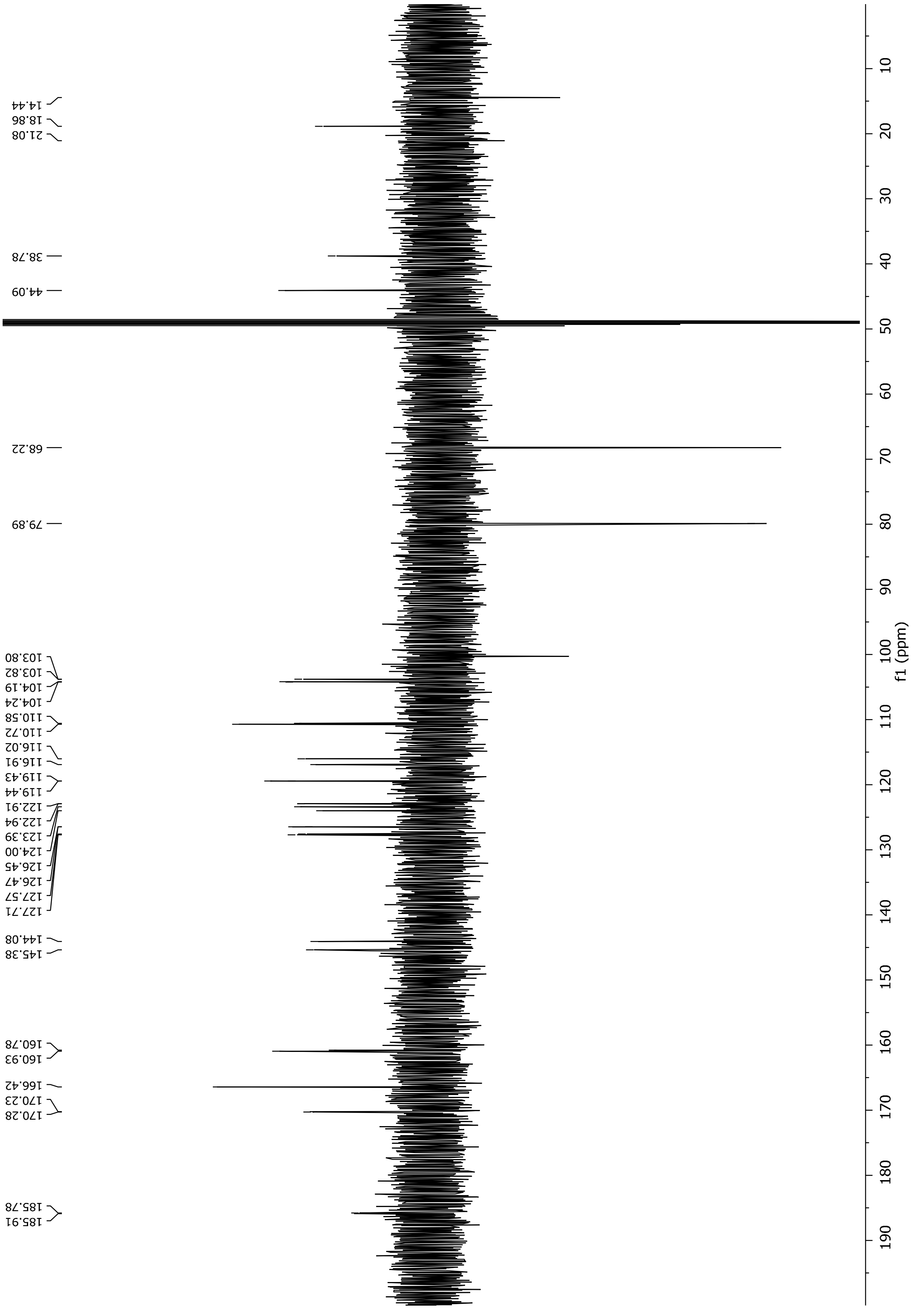

Figure S13. ^{13}C APT NMR spectrum (200 MHz, MeOH-d_3) of monosulfated gymnochrome A (**3**).

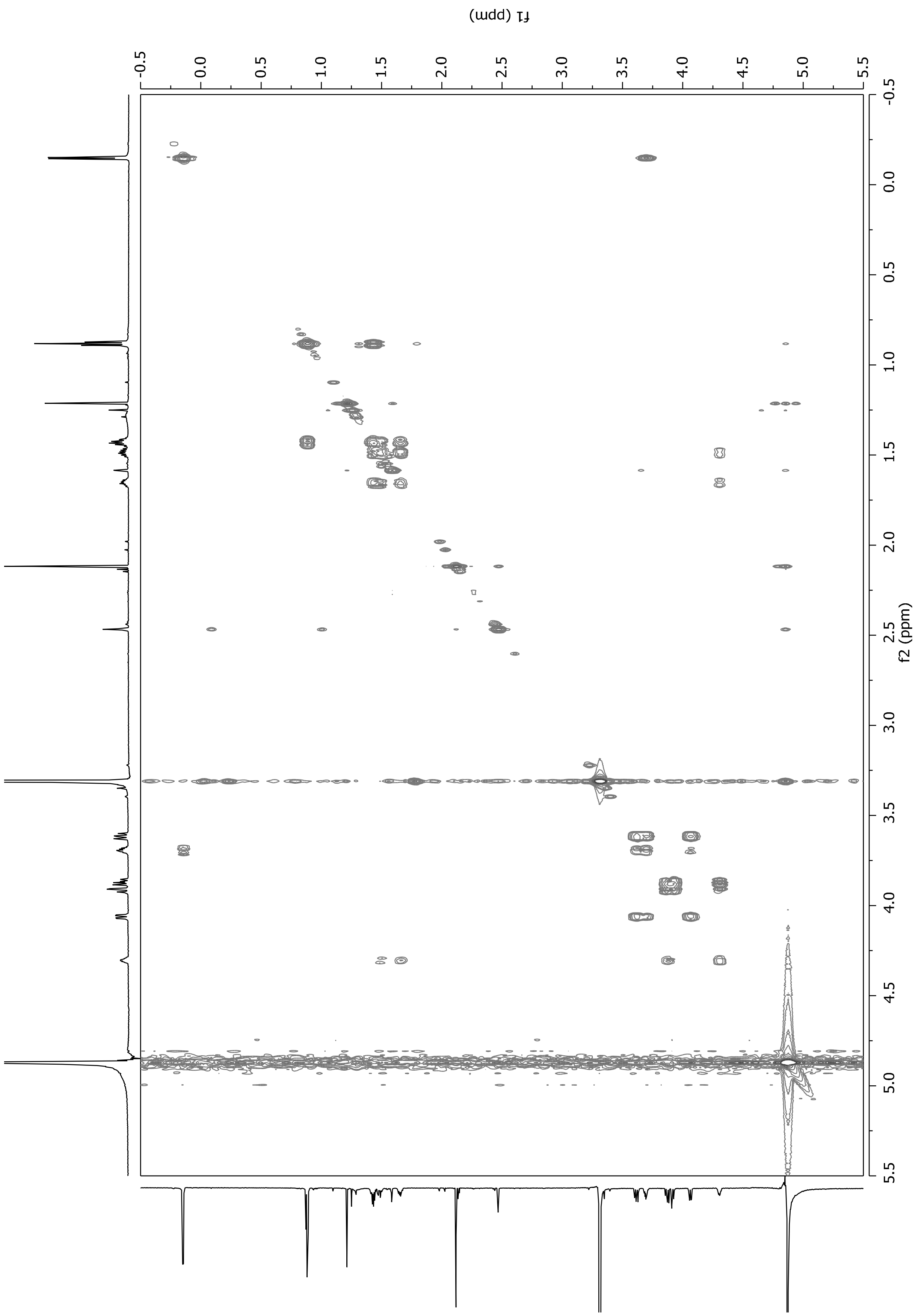

Figure S14. COSY spectrum (800 MHz, MeOH-d_3) of monosulfated gymnochrome A (**3**).

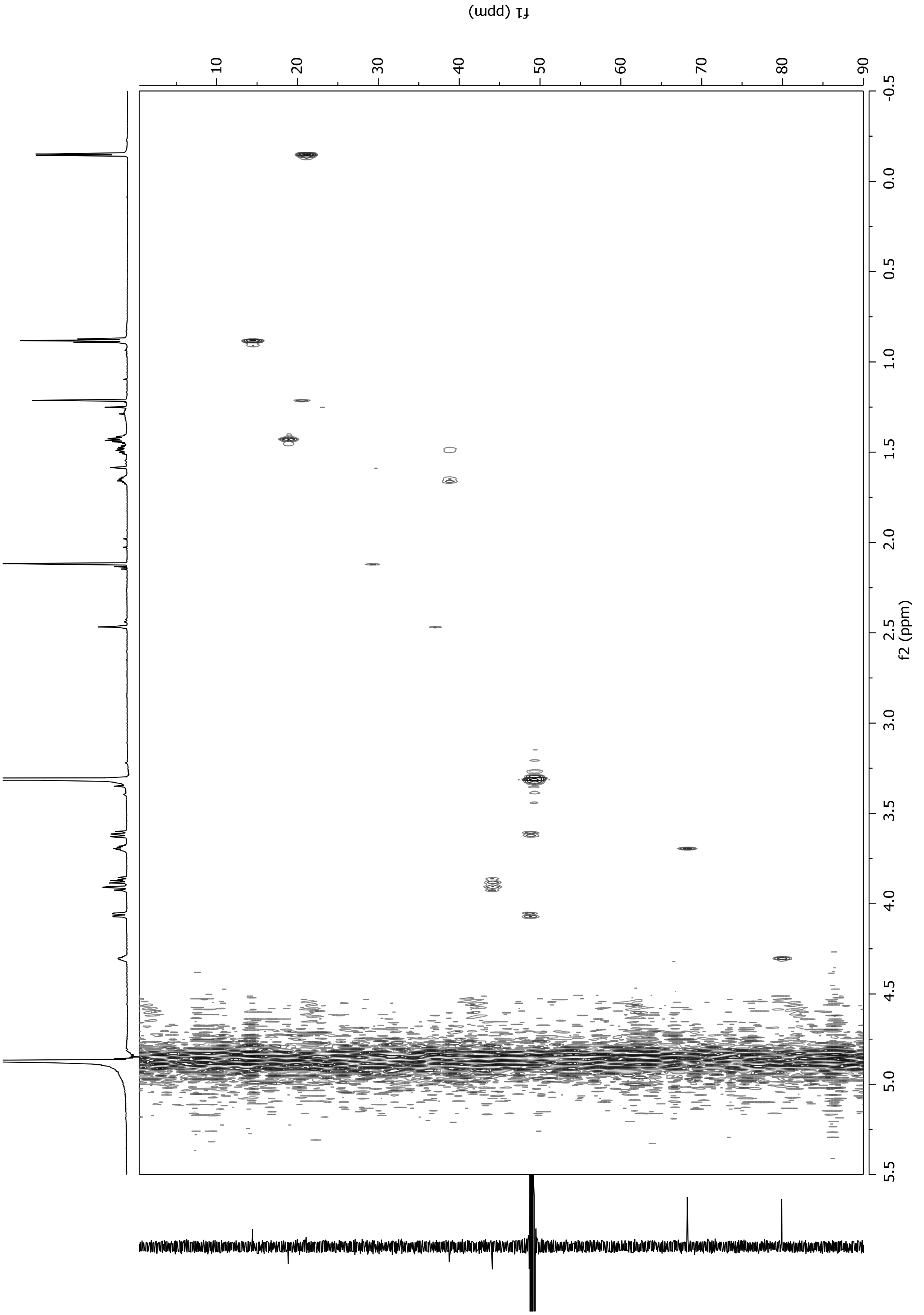

Figure S15. HSQC spectrum (800 MHz, MeOH-d_3) of monosulfated gymnochrome A (**3**).

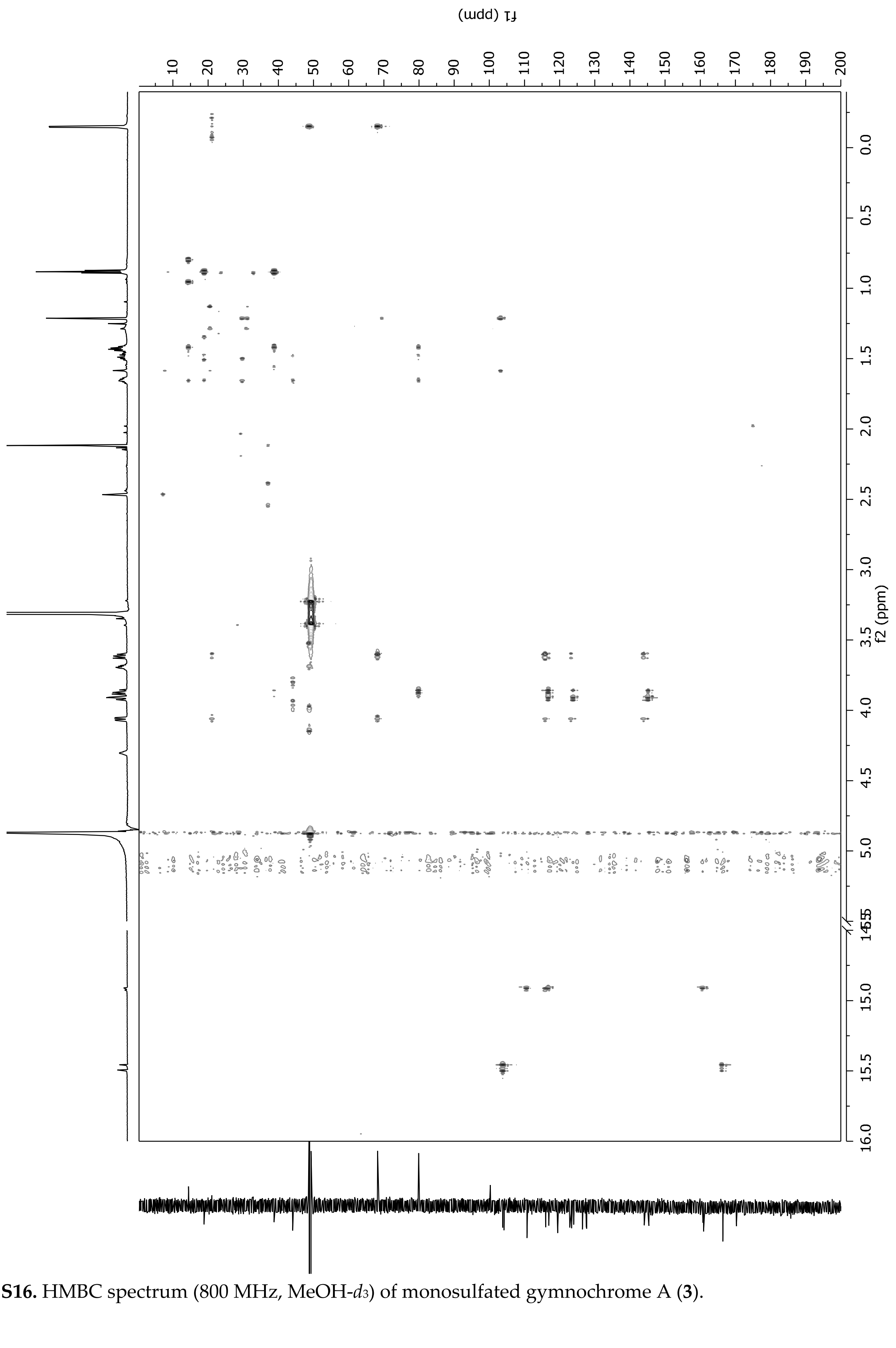

Figure S16. HMBC spectrum (800 MHz, MeOH-d_3) of monosulfated gymnochrome A (**3**).

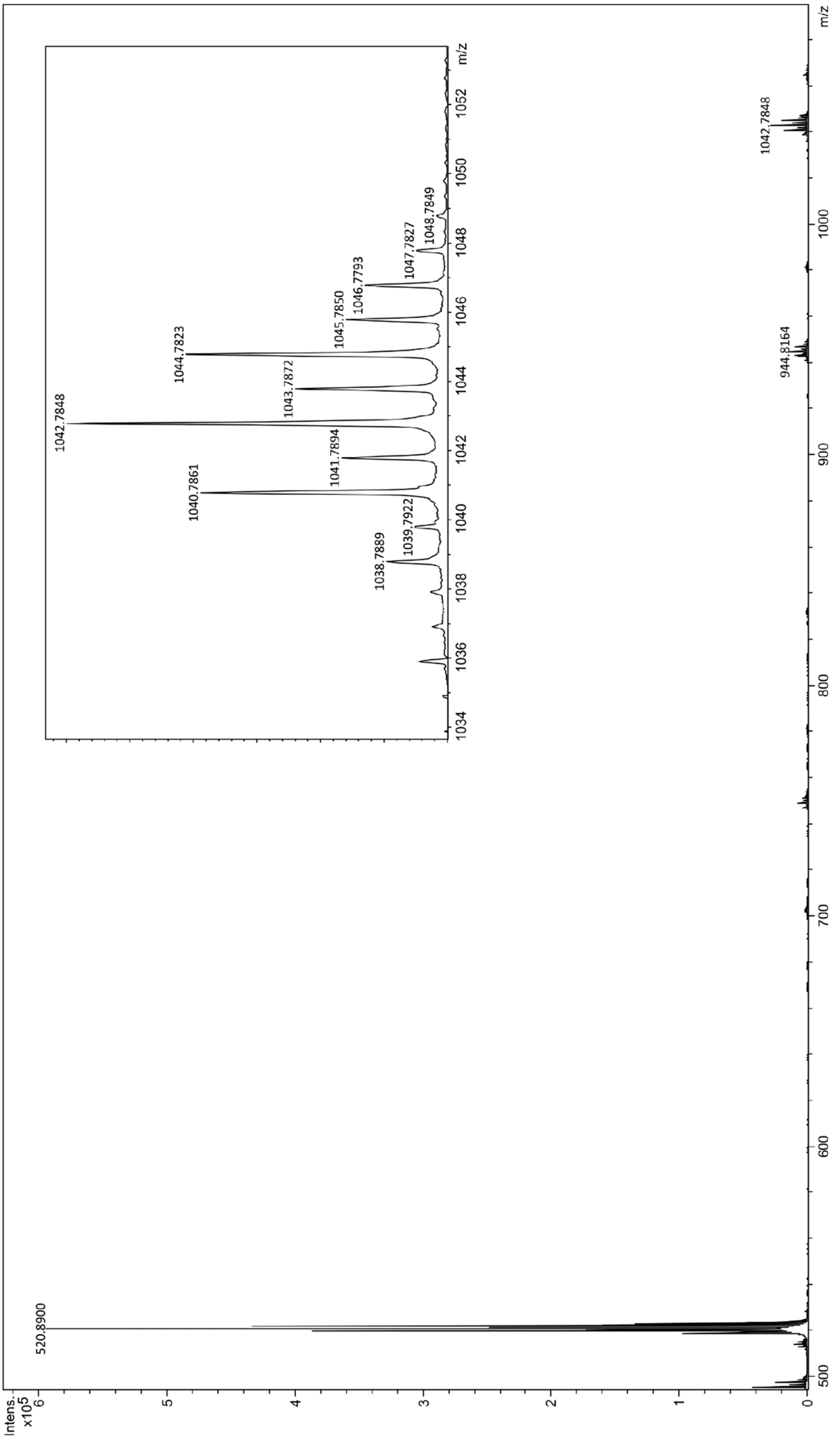

Figure S17. HRESIMS spectrum (negative-ion mode) of monosulfated gymnochrome D (**5**).

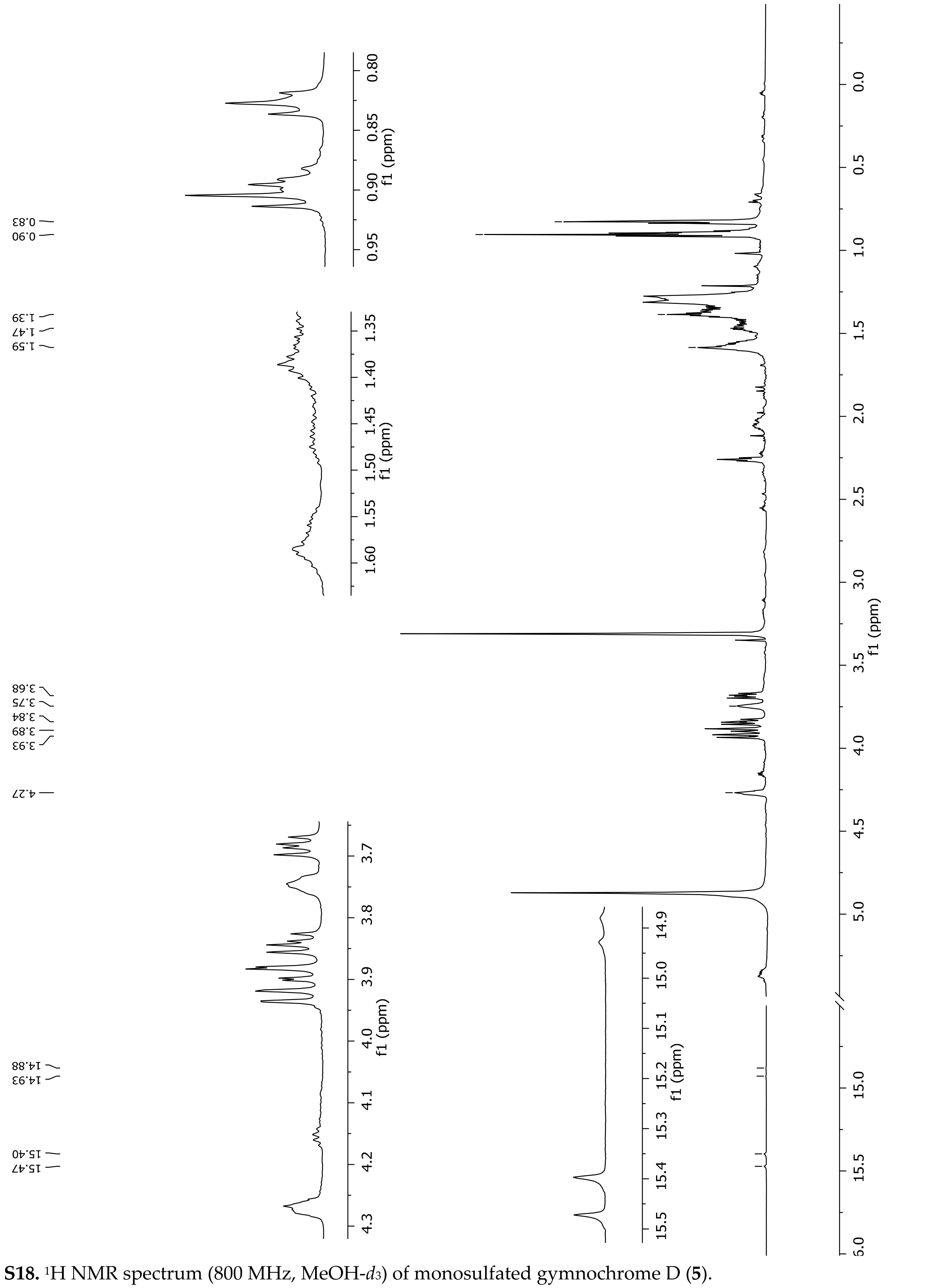

Figure S18. ^{1}H NMR spectrum (800 MHz, MeOH-*d*$_3$) of monosulfated gymnochrome D (**5**).

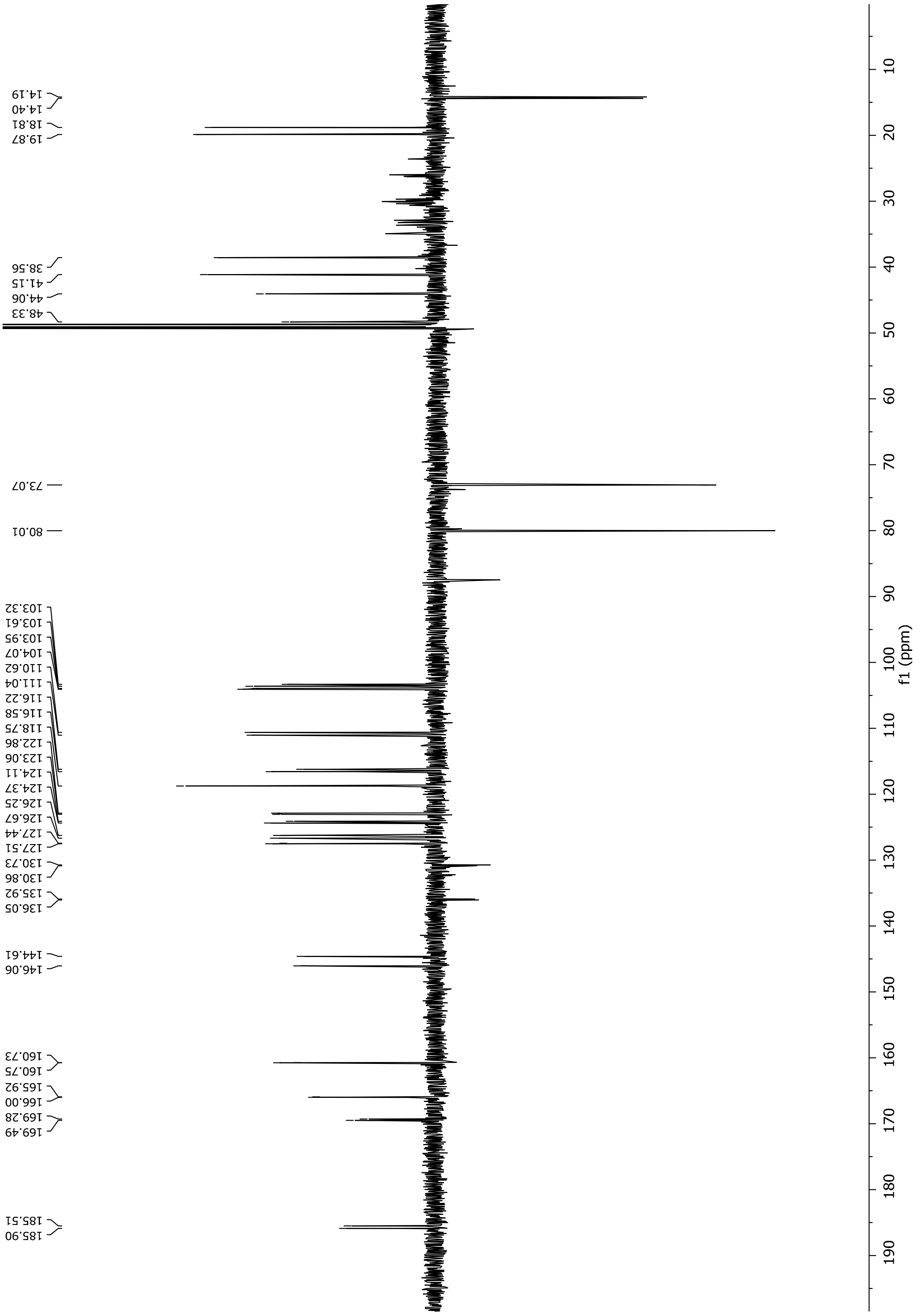

Figure S19. ^{13}C APT NMR spectrum (200 MHz, MeOH-*d*3) of monosulfated gymnochrome D (**5**).

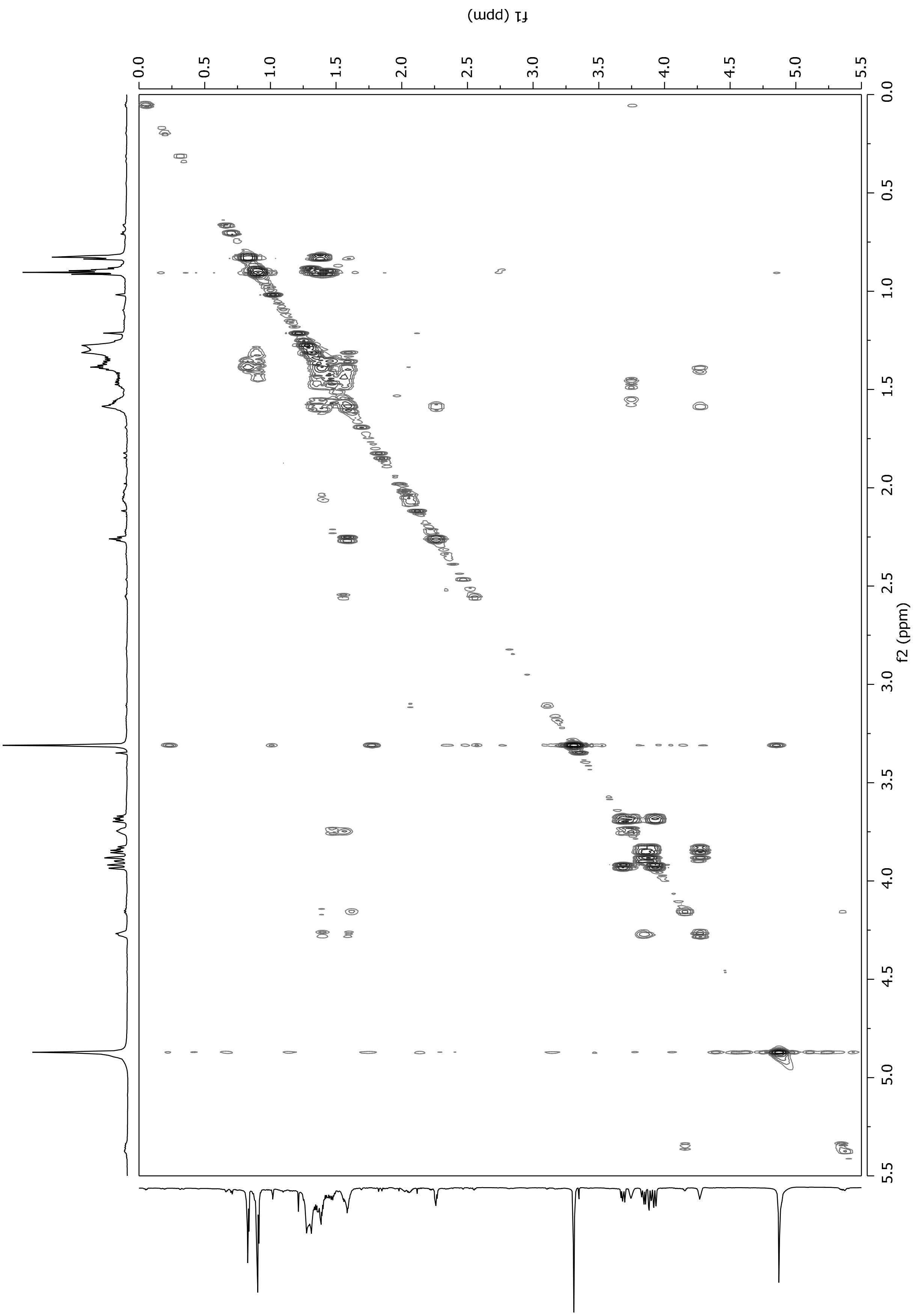

Figure S20. COSY spectrum (800 MHz, MeOH-d_3) of monosulfated gymnochrome D (**5**).

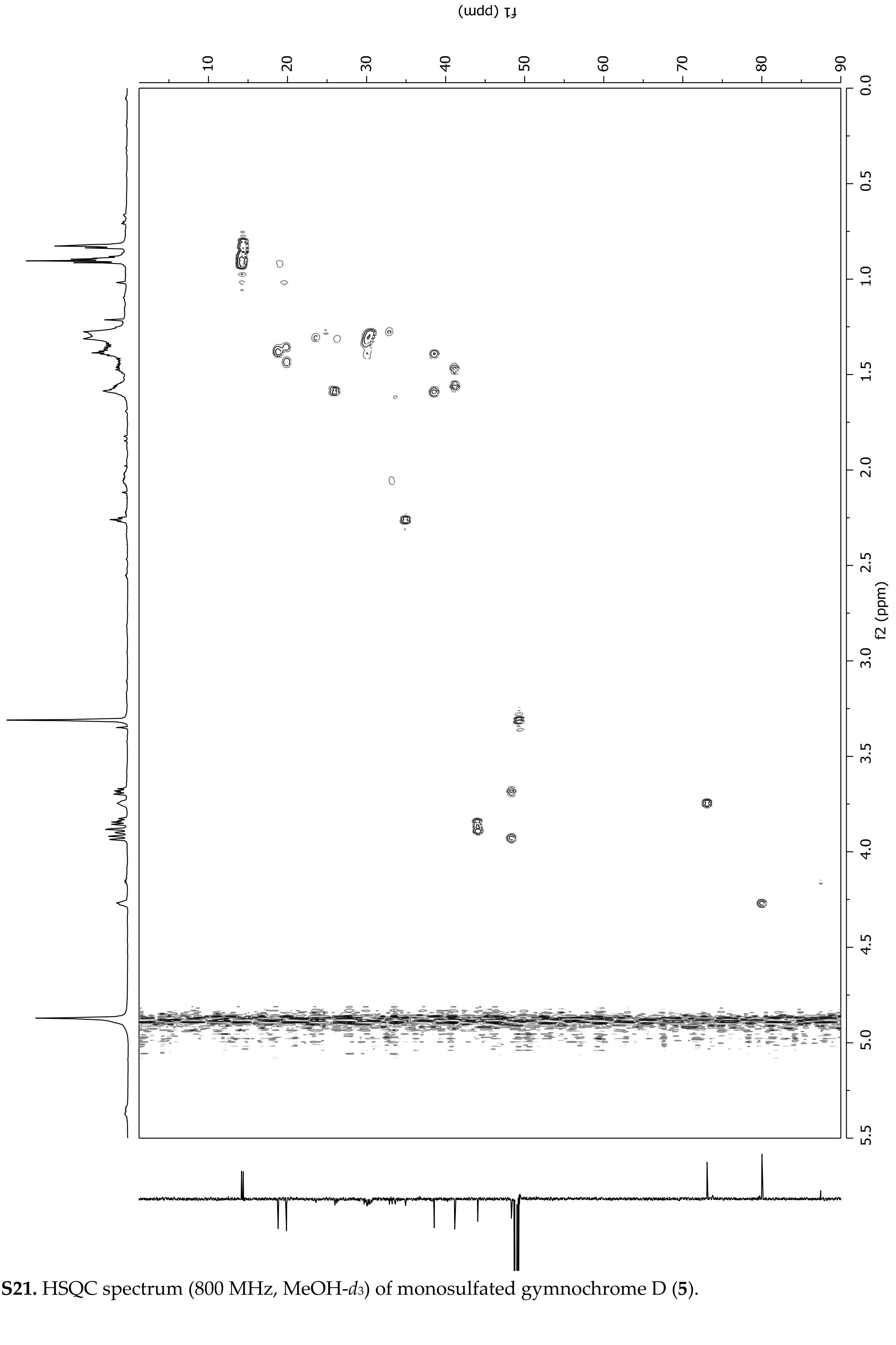

Figure S21. HSQC spectrum (800 MHz, MeOH-d_3) of monosulfated gymnochrome D (**5**).

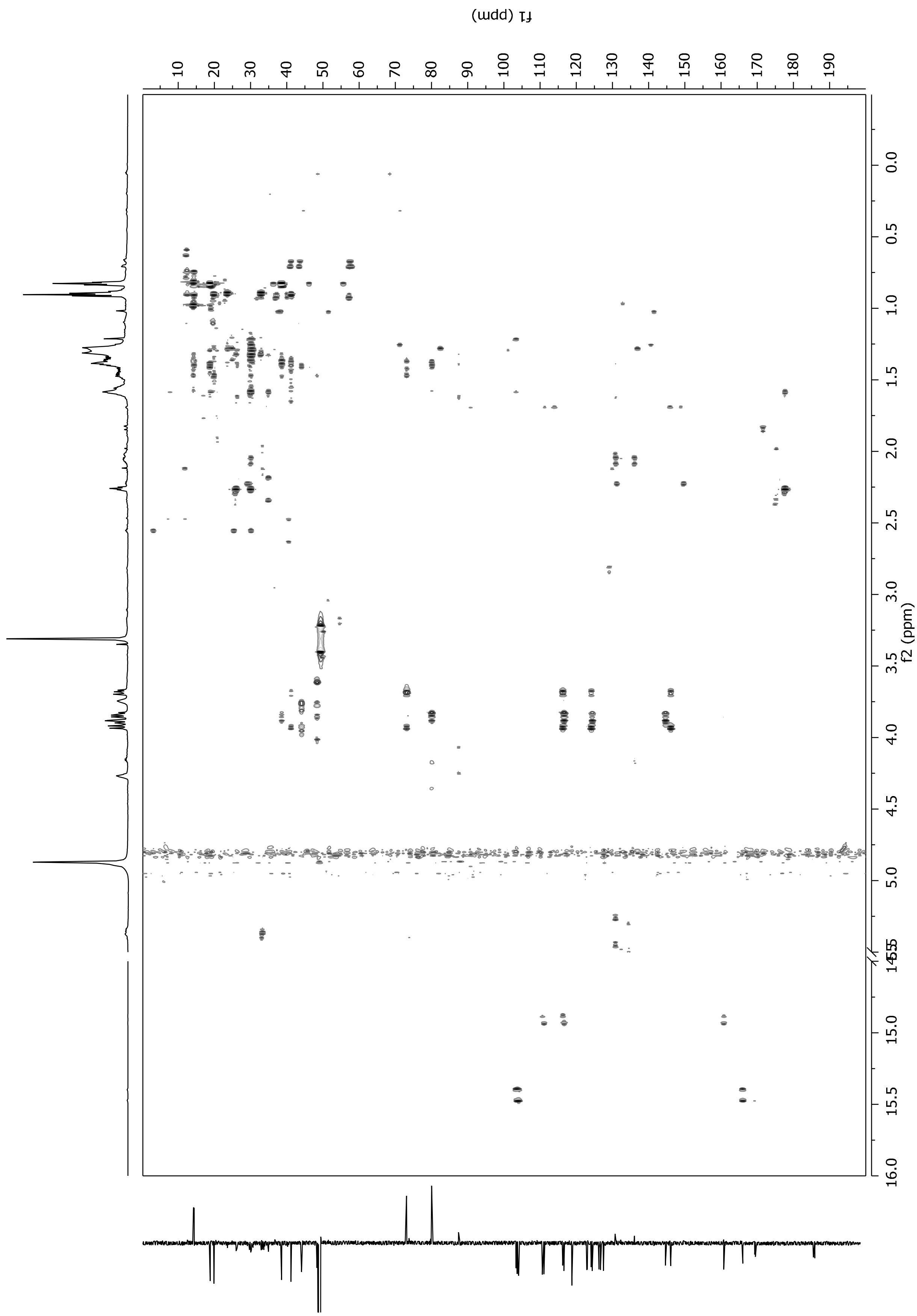

Figure S22. HMBC spectrum (800 MHz, MeOH-*d*3) of monosulfated gymnochrome D (**5**).

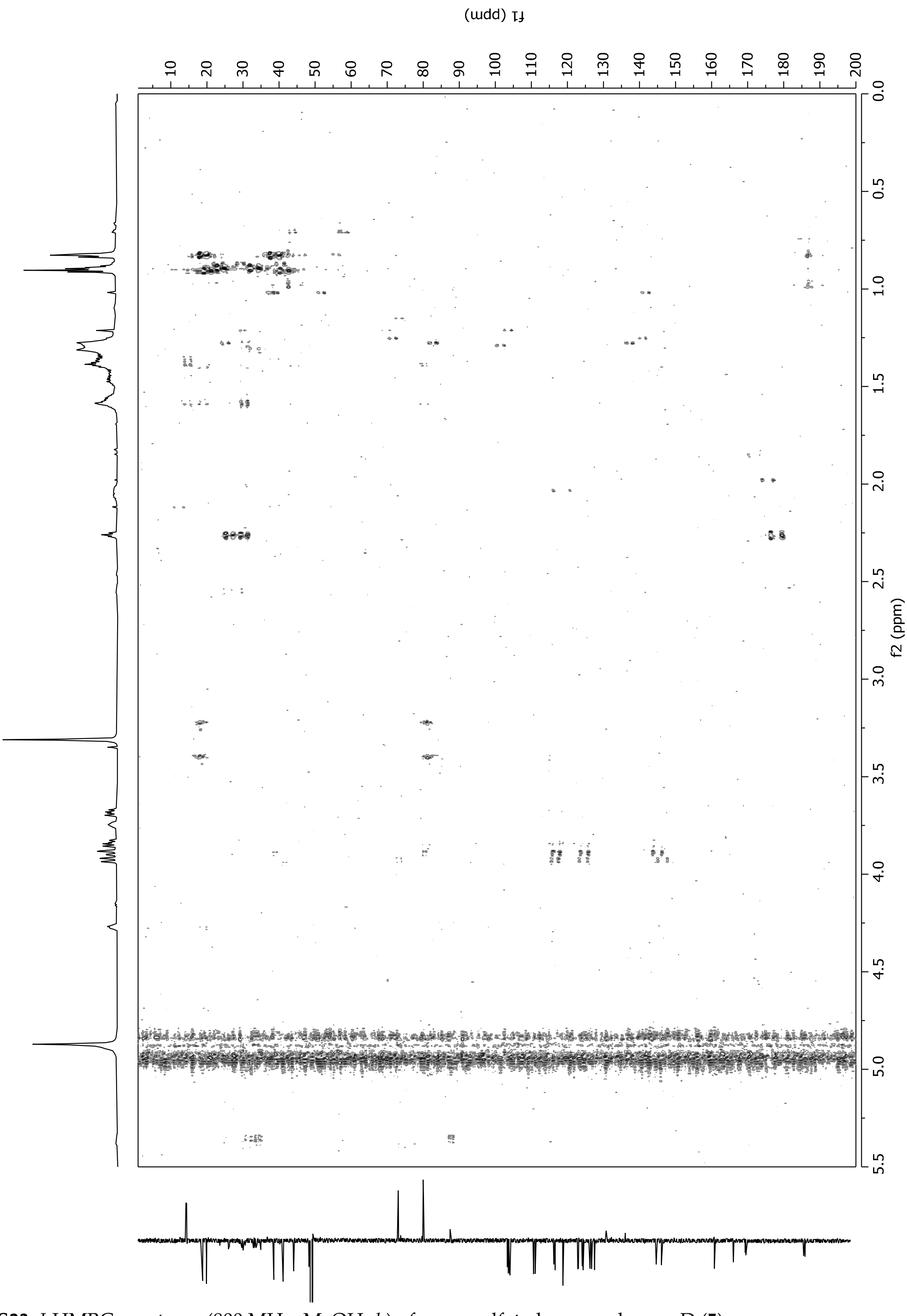

Figure S23. *J*-HMBC spectrum (800 MHz, MeOH-d_3) of monosulfated gymnochrome D (**5**).
